# A point mutation in the FAT domain constitutively increases the kinase activity of Rad3^ATR^ and bypasses the requirement for 9-1–1 phosphorylation to activate the DNA replication checkpoint

**DOI:** 10.1371/journal.pgen.1012213

**Published:** 2026-06-22

**Authors:** Kamal Dev, S. Dean Rider, Balveer Singh, Abhinav Saini, Yong-jie Xu

**Affiliations:** Department of Pharmacology and Toxicology, Boonshoft School of Medicine, Wright State University, Dayton, Ohio, United States of America; Fred Hutchinson Cancer Research Center, UNITED STATES OF AMERICA

## Abstract

Ataxia telangiectasia and Rad3-related (ATR) initiates cell cycle checkpoints to maintain genome integrity in the presence of replication stress or various forms of DNA damage. However, how ATR is activated for checkpoint initiation remains incompletely understood. The canonical model suggests that binding of an ATR-activator protein relieves the autoinhibitory PIKK regulatory domain (PRD) within the kinase domain, thereby activating ATR by granting substrate access to the catalytic centre. To better understand the checkpoint initiation mechanism, we conducted a genetic screen in fission yeast that identified a charge-reversal mutation, E1369K, in the conserved FRAP-ATM-TRRAP (FAT) domain of Rad3, the ortholog of ATR. *In vitro* kinase assays show that the mutation converts Rad3 into a constitutively active form. This allows rescue of the Rad3 kinase signaling defect in cells lacking the phosphorylation of the Rad9-Rad1-Hus1 (9-1-1) complex specifically in the DNA replication checkpoint, not the damage checkpoint pathway. Since the mutation is not in the kinase domain and is away from the PRD, these findings show that, in addition to the canonical mechanism, Rad3 may also be activated allosterically via the FAT domain, a mechanism likely conserved in higher eukaryotes.

## Author summary

ATR is the sensor kinase that activates cell cycle checkpoints in response to DNA replication stress or damage. Although essential for safeguarding the genome, how ATR initiates the checkpoints at the stressed fork or site of damage remains incompletely understood. The current model suggests that binding of an activator protein relieves the autoinhibitory function of the PRD domain within the kinase domain, which activates ATR by granting substrate access to the catalytic centre. To investigate the checkpoint initiation mechanism in fission yeast, we carried out a genetic screen and identified an E1369K mutation in the conserved FAT domain of Rad3, the ortholog of ATR. *In vitro* kinase assays show that the mutation converts Rad3 into a constitutively active form, which suggests that, in addition to the current model, Rad3 may also be activated allosterically via the FAT domain. Interestingly, the mutation can specifically rescue the Rad3 kinase signaling defect caused by phospho-mutations in the Rad9-Rad1-Hus1 complex at the stressed fork, not the DNA damage site. These results provide insights into the checkpoint mechanisms likely conserved in higher eukaryotes.

## Introduction

Eukaryotic cells ensure high-fidelity of DNA replication, repair, and cell division through tightly integrated cell signaling networks that guard genome integrity. Replication stress, whether caused by external genotoxins or internal metabolic imbalances or suppression, can stall or collapse replication forks, posing a serious threat to genome stability [1,2]. Eukaryotic cells manage replication stress mainly through a highly conserved mechanism, the DNA replication checkpoint (DRC), which stabilizes stalled forks, prevents premature mitosis, and promotes replication and repair, ensuring DNA replication is complete before cell division [3]. When the DRC fails, stalled forks become unstable and may collapse into double-strand breaks (DSBs), the most lethal form of DNA damage. DNA damage can also activate the DNA damage checkpoint (DDC) to promote repair while suppressing premature mitosis. Therefore, the DRC and DDC are crucial for maintaining genome integrity. Defects in the DRC and DDC cause chromosomal abnormalities and genome instabilities, which drive cancer development and other genetic disorders [4].

Although eukaryotic cells rely on the checkpoints to manage replication stress, the molecular mechanisms, particularly at the initiation stage, remain incompletely understood. The current model suggests that a set of sensor proteins conserved in all eukaryotes assembles at the perturbed fork or site of damage to initiate the checkpoint signaling [3]. Checkpoint signaling is then relayed through mediator proteins to the effector kinases CHK1 and CHK2, which phosphorylate hundreds of cellular proteins to mediate the checkpoint functions described above. In humans, ataxia–telangiectasia mutated (ATM) activates the checkpoint mainly through CHK2 in the presence of DSBs, whereas ATR activates CHK1 in response to replication stress or various other forms of DNA lesions. For ATR-initiated checkpoint signaling, the replication protein A (RPA)-coated single-strand DNA (ssDNA) binds to ATRIP, the cofactor of ATR, which recruits the ATR-ATRIP complex to the fork or damage site to initiate the checkpoints. The RPA-ssDNA platform also promotes the loading of the Rad9-Rad1-Hus1 (9-1-1) clamp at the 5’ end of the ssDNA/dsDNA junction [5–7]. The loaded 9-1-1, upon phosphorylation by ATR, recruits additional checkpoint proteins, such as TopBP1, carrying an ATR activation domain [8]. The recruited TopBP1 activates ATR and amplifies its kinase signaling. Like TopBP1, Ewing Tumor-Associated Antigen 1 (ETAA1) can also activate ATR both *in vitro* and *in vivo* [9–11]. In budding yeast, three proteins have been identified that possess a Mec1^ATR^-activation domain: Ddc1^Rad9^, the large subunit of the 9-1-1 complex; Dna2, an Okazaki maturation factor on the lagging strand; and Dpb11^TopBP1/Rad4^, which is recruited by phosphorylated Ddc1 and the major Mec1 activator in budding yeast [12–14]. Although structural evidence is still lacking, it is believed that binding of an activation domain activates ATR by relieving the autoinhibitory PRD, which enables substrate access to the catalytic center [11,15,16]. Once activated, ATR activates CHK1, and similarly, Mec1 phosphorylates Rad53^CHK2/Cds1^ in budding yeast.

In the fission yeast *S. pombe,* Rad3^ATR/Mec1^ activates both the DRC and the DDC pathways, whereas Tel1^ATM^ has a minimal checkpoint role. Unlike the human ATR-Chk1 and budding yeast Mec1-Rad53 signaling cascades, Rad3 activates the effector kinase Cds1^CHK2/Rad53^ at the fork and Chk1 at the damage site to activate the DRC and DDC separately. Therefore, examining the phosphorylation of Cds1 or Chk1 can reveal the origins of Rad3 kinase signaling. This promotes an unambiguous description of the checkpoint initiation mechanism. By taking this technical advantage in fission yeast, we carried out a genetic screen to better understand DRC mechanisms. Here, we report the discovery of a missense mutation in Rad3 that specifically bypasses Rad9 phosphorylation in the DRC, not the DDC pathway. Examination of Rad3 kinase activity by *in vitro* and *in-cell* assays reveals that, in addition to the current PRD relief mechanism, Rad3 may also be activated by an allosteric mechanism through the FAT domain.

## Results

### Phosphorylation of Rad9 is required for the activation of Cds1^Chk2/Rad53^, not Mrc1^Claspin^

**Fig 1:**
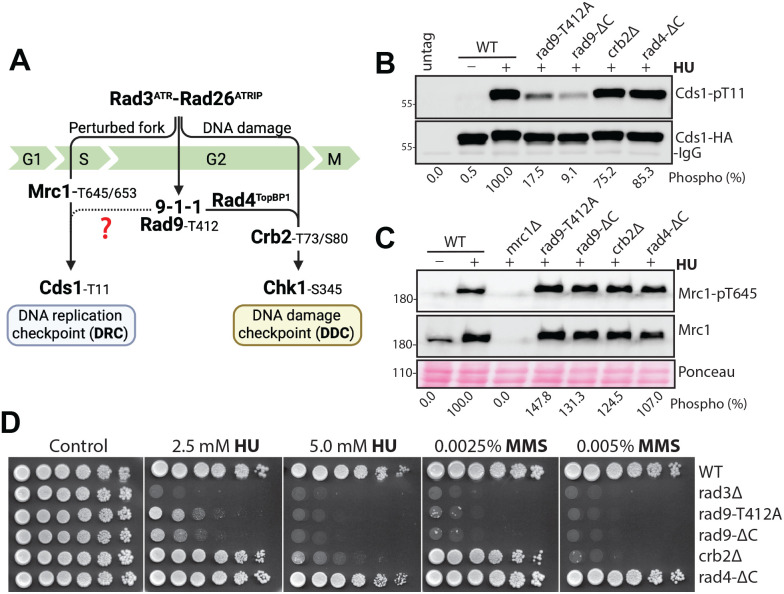
The C-terminus of Rad9 is required for the phosphorylation of Cds1, but not Mrc1. **(A)** Overview of the Rad3^ATR/Mec1^ kinase signaling in the DRC (left) and DDC (right) pathways in fission yeast. Numbers represent Rad3-specific phosphorylation sites. In the DRC, Rad3 phosphorylates Mrc1, which recruits Cds1 to be phosphorylated by Rad3. In the DDC, phosphorylated Rad9 recruits Rad4 to promote Rad3 phosphorylation of Crb2 and Chk1. Rad3 also phosphorylates Rad9 of the Rad9-Rad1-Hus1, or 9-1-1 complex, which is necessary for Rad3 phosphorylation of both Cds1 and Chk1. However, the function of Rad9 phosphorylation in Cds1 activation remains unknown, as indicated by the question mark. **(B)** Rad3 phosphorylation of Cds1 was examined by Western blotting using a phospho-specific antibody in wild-type *S. pombe* and the mutants with the indicated mutation treated with (+) or without (-) 15 mM HU for 3 **h.** Cds1-HA was IPed from whole cell lysates and separated by SDS PAGE for the Western analysis using anti-HA antibodies to detect Cds1 (lower panel). The membrane was stripped and re-blotted with the phospho-specific antibody (upper panel). The phosphorylation bands were quantified and shown below in percentages. **(C)** Rad3 phosphorylation of Mrc1 in wild-type and mutant *S. pombe* with indicated mutations. The cells were treated with HU as in B and fixed in 15% trichloroacetic acid. Whole cell extracts made by using a mini-bead beater were analysed by Western blotting using the antibody against Mrc1-pT645 (top panel). The membrane was stripped, washed overnight, and reblotted with the antibody against Mrc1 (middle panel). A section of the Ponceau S-stained membrane is shown for loading (bottom panel). The quantitation results are shown at the bottom. **(D)** Drug sensitivities of *S. pombe* mutants used in B and C were determined by spot assay. Wild-type and *rad3∆* strains were included as controls. A series of five-fold dilutions of logarithmically grown *S. pombe* were spotted on a YE6S plate or YE6S plates containing HU or MMS at the indicated concentrations. Plates were incubated at 30˚C for 3 days and then photographed.

Rad3^ATR/Mec1^, together with its cofactor Rad26^ATRIP/Ddc2^, is the master sensor kinase of the DRC and DDC pathways in fission yeast (Fig 1A). In the DRC, Rad3 phosphorylates two redundant TQ motifs (T645 and T653) in Mrc1, the mediator of the DRC [17,18]. Phosphorylated Mrc1 recruits the effector kinase Cds1 to be phosphorylated by Rad3 at Cds1-T11. Phosphorylation of Cds1-T11 promotes autophosphorylation of Cds1-T328, which directly activates the effector kinase [19]. Activated Cds1 mediates most biological functions of the DRC. When DNA damage occurs during G2, the longest cell cycle phase in fission yeast, Rad3 phosphorylates Crb2^53 BP1/Rad9^ and Chk1 to activate the DDC [20–22].

Rad3 also phosphorylates T412 at the C-terminus of Rad9 in the independently loaded 9-1-1 clamp. In the DDC, phosphorylated Rad9 recruits Rad4^TopBP1/Dpb11^ (also known as Cut5) [23], which collaborates with phosphorylated Crb2 to recruit Chk1 to be phosphorylated by Rad3. Rad9 phosphorylation is also required for Cds1 activation in the DRC, as the phospho-mutations, *rad9-T412A* and *rad9-∆C*, lacking the entire C-terminus (411–426 aa), abolished Cds1 phosphorylation in the presence of hydroxyurea (HU), a replication stress inducer (Fig 1B) [18]. The mediator Crb2 in the DDC is essential for Chk1 phosphorylation [24]; however, its deletion has minimal impact on Cds1 phosphorylation (Fig 1B). Previous studies suggest that Rad4 carries a Rad3 activation domain in the C-terminus [25]. Deletion of the entire C-terminus (∆498–648 aa) in Rad4, however, did not affect Cds1 phosphorylation (Fig 1B) [26]. We also examined Mrc1 phosphorylation in HU (Fig 1C). In HU-treated wild-type cells, Rad3 phosphorylation of Mrc1 was significantly increased. Since Mrc1 is expressed during the G1/S phase and the activated DRC promotes Mrc1 expression [27,28], the Mrc1 protein levels were also increased in HU. Under similar conditions, Mrc1 phosphorylation was moderately increased or unaffected in *rad9-T412A*, *rad9-∆C*, *crb2∆,* and *rad4-∆C* mutants (Fig 1C), confirming the previous results [18,26]. Consistent with the defect in Cds1 phosphorylation, the spot assay showed that the *rad9-T412A* mutant was sensitive to HU, although the sensitivity was slightly lower than that of *rad3∆* and *rad9-∆C* cells (Fig 1D). Since Rad9 phosphorylation is required for Chk1 phosphorylation, the phospho-mutant was also sensitive to the DNA-damaging agent methyl methane sulfonate (MMS) [18,29]. Both HU and MMS cause replication stress, which is mainly managed by DRC, in collaboration with DDC. The *crb2∆* cells were therefore moderately sensitive to both agents [24]. These results show that Rad9 phosphorylation is necessary for Rad3 phosphorylation of both Cds1 and Chk1, but not for Mrc1. Although the role of Rad9 phosphorylation in Chk1 activation is relatively clear [22,30], its function in promoting Cds1 activation remains unknown (Fig 1A, the question mark).

### Genetic screen identified suppressors of *rad9* phospho-mutants

**Fig 2:**
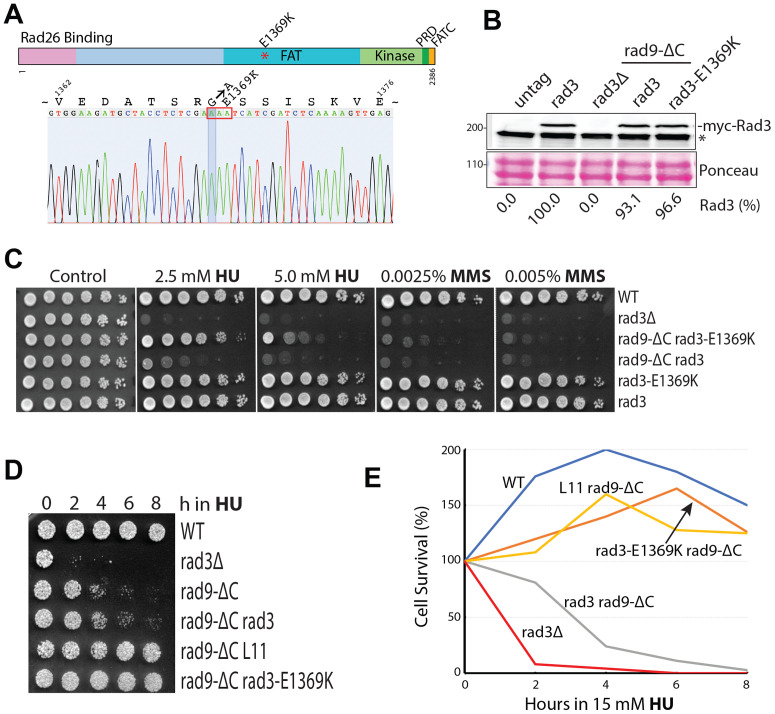
Identification and confirmation of the *E1369K* mutation in Rad3. **(A)** Sanger sequencing confirms the *rad3-E1369K* mutation identified by whole-genome sequencing in the *L11* suppressor. The upper panel shows the domain organization of Rad3. The asterisk indicates the location of the mutation. The bottom panel is the electropherogram showing the G to A nucleotide change. **(B)** The *E1369K* mutation was integrated at the *rad3* genomic locus using the method illustrated in S6 Fig **A.** Integrants of wild-type and the mutant *rad3* were analysed by Western blotting using anti-myc antibody (top panel). Ponceau S staining serves as the loading control (bottom panel). Asterisk indicates a non-specific band. Quantification results are shown at the bottom. **(C)** Drug sensitivities of the *rad3* integrant in *rad9* or *rad9-*Δ*C* background were examined by spot assay as in Fig 1D. **(D)** Acute HU sensitivities of wild type, *rad3∆,* the primary *L11*, and the *rad3* integrants were determined by spot assay. The cells were incubated in YE6S medium containing 15 mM HU. Every 2 h during the HU treatment, a small aliquot of the culture was removed. The cells were washed once and spotted onto a YE6S plate to recover at 30˚C for 3 days. **(E)** Colony recovery assay. The primary *L11* and the *rad3-E1369K* integrant in *rad9-∆C* background were treated with HU, as in **D.** Every 2 h during the treatment, an equal volume of culture was removed, diluted 1000-fold, and spread onto three YE6S plates. The recovered colonies were counted, and the results are presented in percentages.

To identify the missing link between the phosphorylation of Rad9 and Cds1, we mutagenized the genomes of *rad9-T412A* and *rad9-∆C* and screened for suppressors that conferred HU resistance using the strategy illustrated in S1 Fig. With the *rad9-∆C* mutant, we screened a set of K suppressors with increased resistance to HU, MMS, or both (S2 Fig A, top half). Similarly, several L suppressors were screened in *rad9-T412A* (S2 Fig A, bottom half). When Cds1 phosphorylation was examined in the screened suppressors, only *L11* showed robust phosphorylation (S2 Fig B). Since *L11* increased the resistance to MMS, we examined Chk1 activation in the DDC. Surprisingly, Chk1 phosphorylation was not restored in *L11* (S2 Fig C), suggesting a DRC-specific rescuing effect. Since Cds1 phosphorylation depends on phosphorylated Mrc1 [17,18], we examined Mrc1 phosphorylation in HU and found that, while the phosphorylation was unaffected in *rad9-T412A*, it was significantly increased in *L11* containing the *rad9-T412A* mutation (S2 Fig D). Furthermore, the basal level of Mrc1 phosphorylation in untreated *L11* was also higher (Fig 2D), suggesting heightened Rad3 kinase activity in *L11* (see below). To confirm the mutation, we crossed *L11* into *rad9-∆C*. Spot assay showed that *L11* also increased drug resistance in *rad9-∆C* (S3 Fig A). We then extrachromosomally expressed Rad4, Cds1, Rad9, and Suc22, the small subunit of ribonucleotide reductase, under their native promoters in the *L11 rad9-∆C* cells (S3 Fig B). While expression of Rad9 increased drug resistance in *L11 rad9-∆C* to wild-type level as expected, Suc22 increased resistance to HU, not MMS, as Suc22 is the target of HU. Under similar conditions, expression of Rad4 or Cds1 did not change drug resistance in *L11 rad9-∆C*, suggesting that they are not mutated in *L11*. Since other suppressors did not restore Cds1 phosphorylation (S2 Fig B), the following studies were focused on *L11*.

### Genome sequencing of *L11* identified a missense mutation in the FAT domain of Rad3

For genome sequencing, we performed random spore analysis after backcrossing *L11* with the parental *rad9-∆C* strain. Multiple HU-resistant colonies with restored Cds1 phosphorylation were pooled for genome sequencing, which revealed several candidate genes. One of them was *rad3*. Sanger sequencing of the genomic locus identified a single G- > A nucleotide change, causing a charge reversal *E1369K* mutation in Rad3 (Fig 2A). The mutation is in the conserved N-terminal region of the FAT domain (Fig 2A, top panel, and S4 Fig A). The mutated E1369 residue, however, is not highly conserved in budding yeast Mec1 and human ATR (S4 Fig A). Interestingly, the equivalent residue Y1623 in ATR is close to the catalytic centre and distant from the autoinhibitory PRD in the cryo-EM structure of ATR-ATRIP (S4 Fig B) [31–33].

To confirm that the *rad3* mutation restores Cds1 phosphorylation, we tagged Rad3 at the N-terminus with a 10xmyc epitope and expressed wild-type Rad3 and the mutant Rad3 from a vector under the control of its native promoter in the double mutant of *rad3∆ rad9-∆C*. The spot assay showed that while wild-type Rad3 did not affect the drug sensitivity, Rad3-E1369K significantly increased the drug resistance, like the *L11* mutant in *rad9-∆C* (S5 Fig A), although HU resistance was less increased (see below). Western blotting showed that the protein expression level of Rad3-E1369K was similar to wild-type Rad3 (S5 Fig B). When Cds1 phosphorylation was examined, we found that while wild-type Rad3 did not increase the phosphorylation, Rad3-E1369K significantly increased the phosphorylation to a level even slightly higher than in wild-type cells (S5 Fig C). When Mrc1 phosphorylation was examined in these cells (S5 Fig D), the mutant Rad3 increased Mrc1 phosphorylation in HU significantly higher than in wild-type cells.

### Integration of the *rad3-E1369K* mutation at the genomic locus rescues *rad9-∆C* in HU

We then integrated the mutation at the *rad3* genomic locus in wild-type *S. pombe* using the strategy illustrated in S6 Fig A. As a control, wild-type *rad3* was integrated by the same method. Western blotting showed that Rad3-E1369K was expressed in *rad9-∆C* at the wild-type level (Fig 2B). Spot assay showed that while integration of wild-type *rad3* did not rescue *rad9-∆C* as expected, the integration of *rad3-E1369K* increased resistance to HU, but not MMS, in *rad9-∆C* (Fig 2C), which is different from what was observed in *L11* (compare Fig 2C and S2 Fig A). We then compared the *rad3-E1369K* integrant with *L11* on the same drug plate. The integrant was indeed less resistant to HU than *L11*, which showed a higher resistance to both HU and MMS (S6 Fig B).

We have previously reported that chronic HU treatment, such as the spot assay, generates replication stress as well as oxidative stress [34–36]. Acute HU treatment in liquid culture, however, mainly causes replication stress. To investigate the differences between the *rad3-E1369K* integrant and *L11* mutant, we examined acute HU sensitivity. The spot assay showed that when treated with HU for several hours in liquid culture, the *rad3-E1369K* integrant significantly increased HU resistance in *rad9-∆C*, similar to *L11* (Fig 2D). We also performed the colony recovery assay; the *rad3-E1369K* integrant also behaved similarly to the *L11* mutant (Fig 2E). These results suggest that *L11* carries a secondary mutation with a further enhanced HU resistance. To investigate, we performed tetrad dissection of *L11* after backcrossing with *rad9-∆C* and found that while some of the tetrads had two HU-resistant and two HU-sensitive spores, as expected, three tetrads showed three HU-resistant spores (S6 Fig C, green dashed squares) and the levels of HU resistance varied among the spores. This result confirms that *L11* carries a secondary mutation that promotes cell survival, likely in HU-induced oxidative stress, such as the metabolic mutants we have discovered earlier [35,36]. To confirm, we added the antioxidant N-acetyl cysteine to the spot assay and found that it significantly increased the HU resistance of *rad3-E1369K* integrant in *rad9-∆C* (S6 Fig D). Together, these results show that the *rad3-E1369K* mutation rescues *rad9-∆C* from HU-induced replication stress.

### Mrc1 phosphorylation is necessary for Cds1 phosphorylation in *rad3-E1369K*

**Fig 3:**
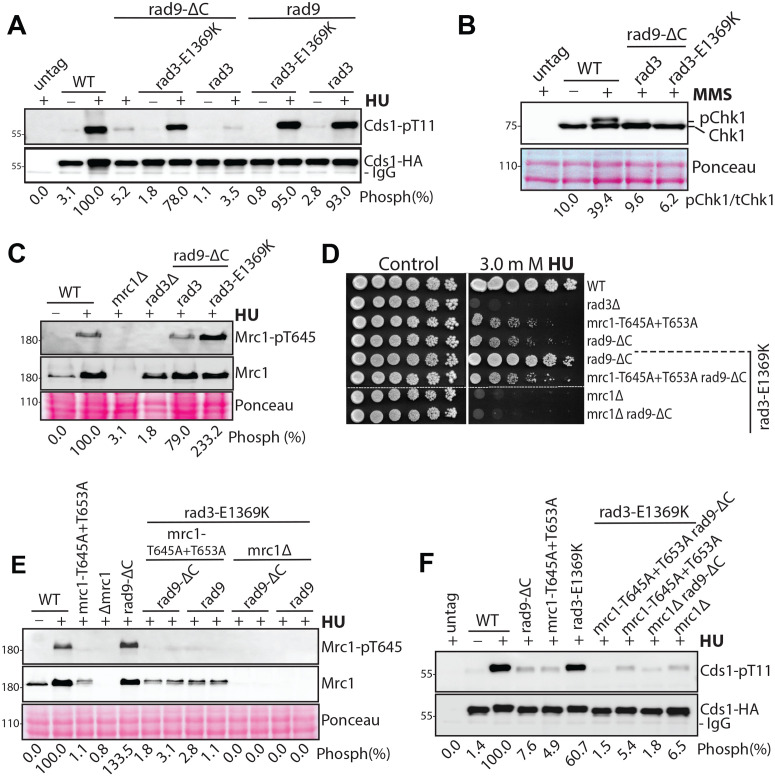
The *rad3-E1369K* mutation restores the phosphorylation of Cds1, not Chk1, in *rad9-∆C.* **(A)** Rad3 phosphorylation of Cds1 in wild-type *S. pombe* and the two *rad3* integrants in *rad9* or *rad9-*Δ*C* background was examined by Western blotting as in Fig 1B. Quantification results are shown at the bottom. **(B)** Chk1 phosphorylation in wild-type and the indicated *rad3* integrant cells was examined by mobility-shift assay. The cells were treated with 0.01% MMS for 90 min. Whole cell lysates were analysed by Western blotting using anti-HA antibody. Chk1 phosphorylation in the upper shifted band was quantified and shown at the bottom as the ratios of phosphorylated Chk1 over total Chk1. **(C)** Rad3 phosphorylation of Mrc1 in wild-type *S. pombe*, the checkpoint mutants *mrc1∆* and *rad3∆,* and the two *rad3* integrants was examined by Western blotting as in Fig 1C. **(D)** The rescuing effect of *rad3-E1369K* in *rad9-∆C* relies on Mrc1 phosphorylation. The HU sensitivities of wild-type and the mutants with the indicated mutations were examined by spot assay. The *mrc1-T645A-T653A* mutation eliminates Rad3-specific phosphorylation on Mrc1. **(E)** Mrc1 phosphorylation was examined by Western blotting in the strains used in **D. (F)** Cds1 phosphorylation was examined in the strains used in **D.**

Using the *rad3-E1369K* integrant, we re-examined Cds1 phosphorylation in *rad9-T412A* or *rad9*-∆C cells. As shown in Fig 3A, HU treatment significantly increased Cds1 phosphorylation in both *rad3* and *rad3-E1369K* integrants in *rad9* cells, as in wild-type cells. However, unlike the *rad3* integrant, which did not increase Cds1 phosphorylation in HU-treated *rad9-∆C*, the *rad3-E1369K* integrant significantly increased Cds1 phosphorylation in *rad9-∆C*, although the level was slightly lower than in wild-type cells. We also re-examined the phosphorylation of Chk1 and Mrc1. In the presence of MMS, Chk1 phosphorylation was not restored in *rad3-E1369K rad9-∆C* cells (Fig 3B), similar to L11 (S2 Fig C). When Mrc1 phosphorylation was re-examined, the phosphorylation was increased in *rad3-E1369K rad9-∆C* more than twofold than in wild-type cells. These results are consistent with the results described for *L11* and confirm that the *rad3-E1369K* mutation bypasses Rad9 phosphorylation to specifically activate Cds1 at the HU-treated fork.

As described above, Mrc1 phosphorylation recruits Cds1 to be phosphorylated by Rad3. Since the *rad3-E1369K* mutation bypasses Rad9 phosphorylation, we then investigated whether it bypasses Mrc1 phosphorylation. We crossed *mrc1∆* or *mrc1-T645A-T653A,* lacking Rad3-specific phosphorylation sites [17,18], into *rad3-E1369K rad9-∆C*. Spot assay showed that *rad3-E1369K* did not rescue *rad9-∆C* in the presence of either *mrc1-T645A-T653A* or *mrc1∆* (Fig 3D). Western blotting for Mrc1 phosphorylation confirmed the strains (Fig 3E). Consistent with the spot assay, phosphorylation of Cds1 was not increased in the *mrc1* mutants (Fig 3F), showing that, unlike Rad9 phosphorylation, the mutant Rad3 still relies on Mrc1 phosphorylation to activate Cds1. We then examined whether the mutation bypasses *rad9∆* and *rad1∆* of the 9-1-1 complex, and the loader *rad17∆* mutant (S7 Fig). The results clearly showed that although it can bypass Rad9 phosphorylation, Rad3-E1369K still needs the loaded 9-1-1 for Cds1 activation.

### Rad3-E1369K overcomes Rad26^ATRIP/Ddc2^ mutations with defects in Rad3 recruitment

**Fig 4:**
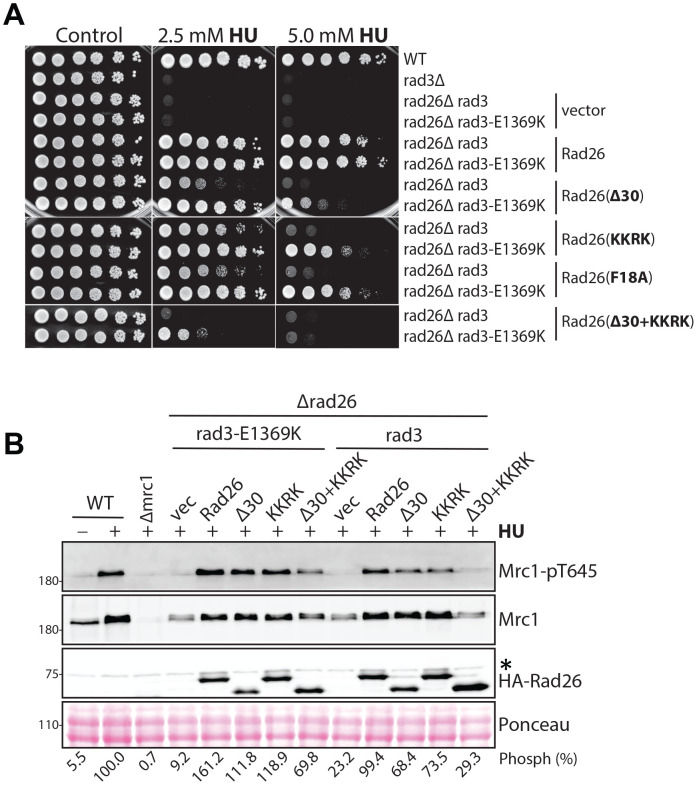
The *rad3-E1369K* mutation partially rescues *rad26* mutants with defects in Rad3 recruitment. **(A)** Wild-type Rad26 and mutant Rad26 with the indicated mutations were expressed on a vector under its native promoter in the *Δrad26 S. pombe* expressing wild-type Rad3 or Rad3-E1369K from the genomic locus. The HU sensitivities of the indicated strains were determined by spot assay. **(B)** Mrc1 phosphorylation in the strains used in (A) was examined by Western blotting. The middle portion of the same membrane was blotted with anti-HA antibody to detect the N-terminally tagged Rad26. The asterisk indicates a cross-reaction material. A section of the Ponceau S-stained membrane is shown as the loading control. Quantification results are shown at the bottom.

**Fig 5:**
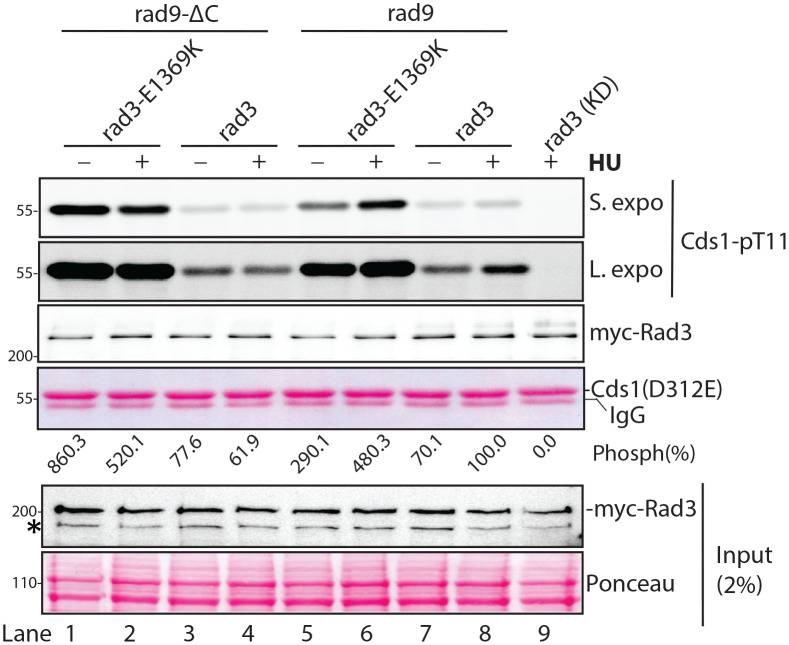
Rad3-E1369K is constitutively active. Wild-type Rad3, Rad3-E1369K, and kinase-inactive Rad3 were IPed from *rad9* or *rad9-∆C* cells treated with (+) or without (-) HU using anti-myc antibody bound to the magnetic Dynabeads. After a brief wash, the IPed Rad3 was incubated with bacterially purified kinase-dead Cds1(D312E) as the substrate to examine the kinase activities as described in Materials and Methods. The samples were incubated for 60 min at 25°C and separated by SDS-PAGE followed by Western blotting using the phosphor-specific antibody against Cds1-pT11 in the bottom part of the blot (top two panels with short or long exposures). The top part of the blot was incubated with anti-myc antibody to detect Rad3 (3rd panel from the top). The Cds1 substrate and the IgG used for the IP were shown by Ponceau S staining (4th panel from the top). The Cds1 phosphorylation bands were quantified and shown in percentages at the bottom. The lower two panels are 2.0% inputs of the IP experiment. Rad3 was detected by Western blotting using an anti-myc antibody. Asterisk indicates a cross-reacting material. A portion of the Ponceau S-stained membrane is shown for the loading (bottom panel).

The main function of Rad26 is to recruit Rad3 to the fork or damage site for checkpoint initiation. Rad26 carries an RPA-binding domain (RBD) in the N-terminus. The RBD cooperates with the KKRK motif in Rad26 for Rad3 recruitment. Eliminating the RBD and the KKRK motif significantly reduced the Rad3 kinase signaling at the fork [37]. To see whether the E1369K mutation bypasses the Rad26 mutants, we deleted *rad26* in *rad3* or *rad3-E1369K* integrants and expressed Rad26 or Rad26 mutants on a vector under the control of its native promoter. Spot assay showed that expression of Rad26 mutants of ∆30, lacking the RBD, or the KKRK mutation, sensitized *rad26∆ rad3* cells to HU as previously reported [37], whereas the same *rad26* mutations did not sensitize *rad26∆ rad3-E1369* significantly (Fig 5A). The *F18A* mutation, known to eliminate the RPA-binding activity [37], sensitized *∆rad26 rad3,* but not much in *∆rad26 rad3-E1369K* cells. Expressing Rad26 with the combined mutation *∆30 + KKRK* significantly sensitized both *∆rad26 rad3* and *∆rad26 rad3-E1369K* cells, although *∆rad26 rad3-E1369K* cells also showed enhanced cell survival (Fig 4A).

Mrc1 is a nonessential replisome protein. Once Rad3 is recruited to the fork, it phosphorylates Mrc1 without the need for other known checkpoint proteins (Fig 1) [18]. To investigate how *rad3-E1369K* rescues the *rad26* mutants, we examined Mrc1 phosphorylation (Fig 4B). As previously reported, all *rad26* mutants were expressed at or near the wild-type levels (Fig 4B, 3^rd^ panel from the top) [37]. In *rad26∆ rad3* cells, expression of Rad26 with *KKRK* or *∆30* mutations mildly or moderately reduced Mrc1 phosphorylation, whereas the combined *∆30 + KKRK* mutation almost eliminated Mrc1 phosphorylation in HU. The same Rad26 mutations, when expressed in *rad26∆ rad3-E1369K* cells, did not reduce Mrc1 phosphorylation significantly as in *rad26∆ rad3* cells. Together, these results show that the *rad3-E1369K* mutation significantly rescues *rad26* recruitment mutants by increasing Mrc1 phosphorylation, although it still relies on Rad3 recruitment for the phosphorylation.

The mutated residue E1369 resides in a short SRESS motif of Rad3. AlphaFold modelling of the Rad3-Rad26 heterodimer showed that the SRESS motif forms a small loop between two α helices, and the charge-reversal mutation converts the loop into an α helix (S8 Fig A). The conformational change impacts Rad26, enabling the N-terminus of Rad26 with multiple acidic residues to bind to the mutated region of Rad3 (S8 Fig A, right panels). This raises a possibility that the mutation may enhance Rad26 binding to Rad3 and thus increase Rad3 recruitment, leading to more robust signaling. To investigate this possibility, we performed a co-IP experiment. The results showed that the mutation did not increase the co-IP of Rad3 with Rad26 (S8 Fig B). Since Rad26 binds to Ssb1 [37], the large subunit of RPA, Ssb1 was also co-IPed with Rad26 in *rad3-E1369K* at a level similar to that in wild-type cells. We then performed the DNA pull-down assay (S8 Fig C) [38]. The results showed that Rad3, Rad26, and the Rad26 mutants were pulled down similarly in *rad3-E1369K* as in wild-type *rad3* cells. These results ruled out the possibility that the mutation increases Rad3 recruitment, leading to the increased phosphorylation of Mrc1 and Cds1 in HU-treated *rad9-∆C* cells.

### The *E1369K* mutation constitutively increases Rad3 kinase activity

The higher level of Mrc1 phosphorylation observed in Fig 3C suggests that the mutation may increase Rad3 kinase activity. To investigate, we IPed Rad3 from the strains used in S8 Fig C for an *in vitro* kinase assay using purified kinase-inactive Cds1(D312E) as substrate (S9 Fig) [26]. After the kinase reaction, the samples were analysed by Western blotting to detect Rad3 (3^rd^ panel from the top), the co-IPed Rad26 (2^nd^ panel), and phosphorylated Cds1 (top panel). The results clearly showed that Rad3-E1369K had a significantly higher kinase activity than wild-type Rad3 in cells expressing Rad26 or the Rad26 mutants. The kinase activity of Rad3-E1369K is dependent on Rad26, as only background kinase activity was detected in the vector control.

To further investigate the increased kinase activity of Rad3-E1369K, we IPed Rad3 and Rad3-E1369K from *rad9* or *rad9-∆C* cells treated with or without HU. The kinase-dead Rad3(D2249E) was IPed in parallel, which showed no detectable kinase activity on Cds1 substrate (Fig 5, lane 9). In the absence of HU, wild-type Rad3 showed a basal kinase activity in *rad9* and *rad9-∆C* cells (Fig 5, lanes 3 and 7), while Rad3-E1369K significantly increased kinase activity several-fold in *rad9-∆C* and *rad9* cells (compare lane 1 with lane 3, and lane 5 with lane 7). Interestingly, the kinase activity of Rad3-E1369K was significantly lower in *rad9* cells (compare lane 1 with lane 5). When treated with HU, the kinase activity in wild-type Rad3 did not increase in *rad9-∆C* and *rad9* cells (lanes 4 and 8), suggesting that the HU-activated Rad3 inside the cells, once IPed, returned to the inactive state. However, Rad3-E1369K further increased the kinase activity in *rad9* cells (lanes 5 and 6), but not *in rad9-∆C* cells (lanes 1 and 2), suggesting that the Rad9 C-terminus may suppress the kinase activity of Rad3-E1369K (see Discussion). Together, the results in S9 Fig and Fig 5 clearly showed that the E1369K mutation converts Rad3 into a constitutively active form, and the Rad9 C-terminus may modulate Rad3 kinase activity in both positive and negative manners.

### The Mec1-F2244L equivalent mutation F2250L in Rad3 does not rescue *rad9-∆*C in fission yeast

**Fig 6:**
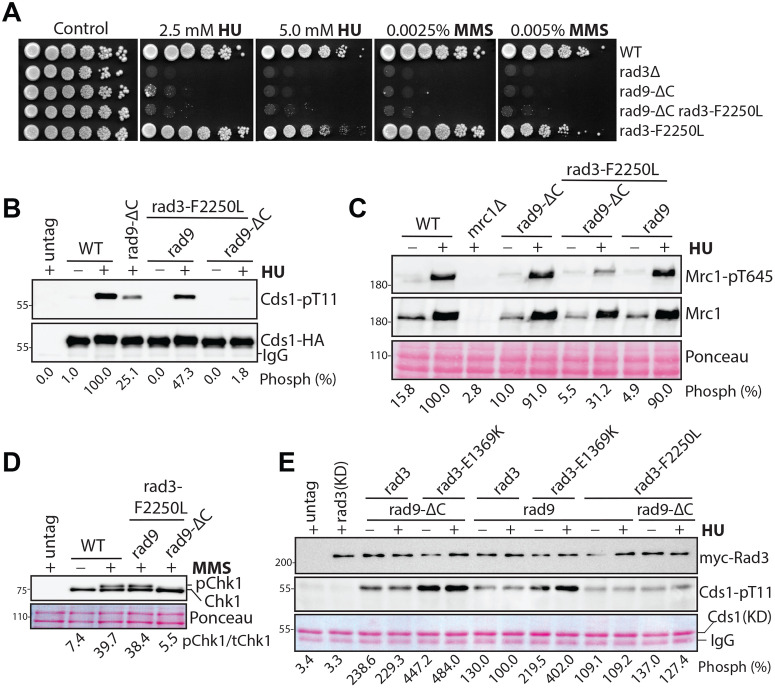
The *Mec1-F2244L* equivalent mutation *F2250L* in Rad3 mildly reduces Rad3 kinase activity *in vitro* and in HU-treated cells. **(A)** Drug sensitivities of wild-type and the *rad3-F2250L* integrant in *rad9* or *rad9-∆C* background were examined by spot assay. **(B)** Rad3 phosphorylation of Cds1 in the strains used in A was examined by Western blotting as in Fig 1B. **(C)** Rad3 phosphorylation of Mrc1 was examined by Western blotting using the phospho-specific antibody (top panel) and anti-Mrc1 antibody (middle panel) as in Fig 1C. A section of the Ponceau S-stained membrane is shown for loading. **(D)** Chk1 phosphorylation in MMS-treated *rad3-F2250L* in *rad9* or *rad9-∆C* background was examined as in Fig 3B. **(E)** Wild-type Rad3, Rad3-E1369K, and Rad3-F2250L were IPed from *rad9* or *rad9-*Δ*C* cells treated with (+) or without (-) HU. The kinase activity of the IPed Rad3 was examined using the Cds1(KD) substrate as in Fig 5.

A recent study in budding yeast identified an F2244L mutation in the DFD motif of the kinase domain in Mec1^ATR/Rad3^ that increases basal kinase activity 10–20-fold *in vitro* and promotes survival of cells lacking all three activators in HU and the genotoxin 4-nitroquinolin 1-oxide [16]. The F2244 residue in Mec1 is conserved in the DFN motif of both Rad3 and ATR (S4 Fig A). We made the equivalent mutation, F2250L, in Rad3 and integrated it at the genomic locus using the same method as for *rad3-E1369K*. After confirming the integration by colony PCR, Western blotting, and sequencing, we crossed the *rad3-F2250L* mutation into *rad9-∆C* to investigate whether it could rescue the *rad9* phospho-mutant, as *rad3-E1369 K* does. The drug sensitivity assay clearly showed that the mutation did not rescue *rad9-∆C*, and the mutation alone minimally sensitized *S. pombe* to HU and MMS (Fig 6A). Western blotting showed that the *rad3-F2250L* mutation did not increase Cds1 phosphorylation in HU-treated *rad9-∆C* cells (Fig 6B). Instead, it moderately reduced Cds1 phosphorylation in *rad9* and eliminated the phosphorylation in *rad9-∆C* cells. The mutation did not affect Mrc1 phosphorylation in HU-treated *rad9* cells, but reduced the phosphorylation in HU-treated *rad9-∆C* cells (Fig 6C). The *rad3-F2250L* minimally affected Chk1 phosphorylation in MMS-treated *rad9* and deletion of the Rad9 C-terminus eliminated the phosphorylation as in *rad3-E1369K* cells (Fig 6D). These results show that the *rad3-F2250L* mutation did not increase the kinase activity of Rad3 as in Mec1. Interestingly, the C-terminus of Rad9 can significantly stimulate the kinase activity of Rad3-F2250L in Mrc1 phosphorylation to nearly the wild-type level in the presence of HU. We then examined the kinase activity of Rad3-F2250L *in vitro*. Western blotting showed that the protein level of Rad3-F2250L was similar to that of Rad3 or Rad3-E1369K (Fig 6E, top panel). Like Fig 5, Rad3-E1369K showed a higher kinase activity than Rad3 in both *rad9* and *rad9-∆C* cells in the absence or presence of HU. Under similar conditions, Rad3-F2250L behaved like wild-type Rad3, although the basal kinase activity was slightly lower, which is consistent with the reduced Cds1 phosphorylation in HU-treated *rad9* and *rad9-∆C* cells (Fig 6B).

## Discussion

The cryo-EM structures of human ATR-ATRIP and budding yeast Mec1-Ddc2 suggest a common feature: the kinase domain is autoinhibited by the PRD via steric hindrance of substrate access [15,32,33]. Although structural evidence is still lacking, it has been proposed that binding of an activation domain to the PRD relieves the autoinhibition, allowing substrate access and realignment of catalytic residues [3]. This activates ATR and Mec1 to initiate the kinase signaling cascades, which, depending on the severity of stress or damage, can be further amplified or suppressed to fine-tune the checkpoint signaling. Consistent with this model, three proteins have been identified in budding yeast that carry the Mec1-activation domain: Ddc1^RAD9^, Dna2, and Dpb11^TopBP1/Rad4^ [12,13,39]. Among them, Dpb11 is the main Mec1 activator in response to replication stress or DNA damage and is recruited by phosphorylated Ddc1^Rad9^ of the 9-1-1 complex. In *Xenopus* and humans, TopBP1 and ETAA1 have been discovered to carry the ATR-activation domain [8,9,11,31].

Our genetic screen has identified an *E1369K* mutation in Rad3, the *S. pombe* ortholog of ATR and Mec1. The mutation restored Cds1 phosphorylation in a Mrc1 phosphorylation-dependent manner and cell survival of *rad9* phospho-mutants in HU (Figs 2 and 3). The *in vitro* kinase assay, together with the in cell data, shows that the mutation converts Rad3 into a constitutively active form, which bypasses the requirement for Rad9 phosphorylation and partially rescues Rad26 mutations defective in Rad3 recruitment. This Rad3 mutation, therefore, raises several interesting questions that require further investigation:

### The activation mechanism of Rad3^ATR/Mec1^

Rad3 robustly phosphorylates Mrc1 in HU without the need for Rad9^Ddc1^ phosphorylation, the C-terminus of Rad4^TopBP1/Dpb11^ (Fig 1C), and even the 9-1-1 complex [18], suggesting that this kinase activity of Rad3 does not require an activator in fission yeast. The increased phosphorylation of Mrc1 and Cds1 by Rad3-E1369K in HU-treated *rad9-∆C* cells (Fig 3A and C) suggests that the charge reversal mutation may increase the binding or proper repositioning of the substrates Mrc1 and Cds1 for more efficient phosphorylation by Rad3, similar to that in mTORC1 and mTORC2 [40,41]. A recent study in budding yeast showed that the F2244L mutation in the DFD motif converts Mec1 into a constitutively active form [16]. However, the equivalent mutation in Rad3 did not rescue *rad9-∆C* cells and mildly reduced Rad3 kinase activity *in vitro* and in HU-treated cells (Fig 6), suggesting that the function of the F2244 residue in the Mec1’s DFD motif may not be conserved in Rad3. The first Asp residue of the DFD motif in the activation loop of Mec1 is absolutely conserved for coordinating with Mg^2+^, whereas the FD residues are less conserved as LG, FN, or FG in PIKKs. The F2244L mutation in Mec1 increases its ATPase activity. However, this increased activity is less efficiently coupled with the phosphor-transfer to the substrate, or kinase activity of the mutant Mec1 [16]. Whether the equivalent mutation F2250L in the DFN motif of Rad3 further decouples the two catalytic processes is unclear and needs further examination. Nonetheless, several lines of evidence support an allosteric activation mechanism in Rad3. First, the E1369K mutation is located in the FAT, not the kinase domain of Rad3. Although not conserved, the equivalent residue Y1623 in ATR is located near the catalytic center, but away from the PRD (S4 Fig B). ATR also autophosphorylates the T1989 residue in the FAT domain to regulate its kinase activity [42,43]. Moreover, autophosphorylation in or near the FAT domain of ATM and DNA-PKcs, two other PIKKs essential for checkpoint signaling and repair of DSBs, respectively, can also regulate their functions [44–47]. Second, the E1369K mutation creates a constitutively active form of Rad3 as demonstrated by the *in vitro* kinase assay (Figs 5, 6E, and S9). Third, as mentioned above, the robust phosphorylation of Mrc1 by Rad3 occurs in HU-treated *rad9-∆C* and *rad4-∆C* cells (Fig 1C), lacking the potential activators Rad9 and Rad4. Rad3-E1369K also hyperphosphorylates Mrc1 in HU-treated *rad9-∆C* cells (Fig 3C). Similarly, colocalization of Mrc1 and Ddc2 in budding yeast by tethering them at an undamaged chromosomal locus allows Mec1 phosphorylation of Rad53 in the absence of all three activators [48]. Fourth, the activated Rad3 in HU-treated cells, once IPed, returned to inactive status *in vitro* (Fig 5). Finally, the constitutively active Rad3-E1369K phosphorylates Cds1, not Chk1, in *rad9* phospho-mutants (Fig 3A and B), suggesting that proper positioning of Chk1 is necessary for the phosphorylation by Rad3. It is likely that Rad3 can be activated by two mechanisms, one through binding to a Rad3-activator protein that remains to be identified in *S. pombe* and the other through allosteric regulation via the FAT domain or the HEAT repeats [49], allowing fine-tuning of its kinase signaling in the context of stress management or damage being actively repaired. It would be interesting to investigate whether the allosteric mechanism revealed by the E1369K mutation occurs under physiological conditions. Nevertheless, further biochemical and structural studies may provide more insights into the activation mechanisms of Rad3 and, potentially, for other PIKKs.

### The positive and negative regulatory roles of the Rad9^Ddc1^ C-terminus

That the constitutively active Rad3-E1369K bypasses *rad9-∆C* to phosphorylate Cds1 suggests that the main function of the Rad9 C-terminus is to promote Rad3 kinase activity. Since Mrc1 is hyper-phosphorylated in *rad3-E1369K* in *rad9-∆C* (Fig 3C), the hyperphosphorylation may increase the recruitment of Cds1, leading to the increased Cds1 phosphorylation in HU (Fig 3A). The dependency of Mrc1 phosphorylation on the increased Cds1 phosphorylation in *rad3-E1369K* supports this possibility (Fig 3D-F). Alternatively, defective Cds1 phosphorylation in *rad9-∆C* promotes Mrc1 phosphorylation, leading to its hyperphosphorylation in *rad3-E1369K,* but not in *rad3* cells. This notion is consistent with the canonical role of Rad9 C-terminus in stimulating Rad3 kinase signaling. In budding yeast, the C-terminus of Ddc1^Rad9^ carries a Mec1-activation domain [13], and the phosphorylated Ddc1 C-terminus can further recruit the major Mec1 activator protein Dpb11 [14,39]. Similarly, the phosphorylated Rad9 C-terminus recruits Rad4 in fission yeast and TopBP1 in mammalian cells [23,29]. Consistent with this positive role of the Rad9 C-terminus, less Cds1 phosphorylation was observed in the double mutant of *rad9-∆C rad3-E1369K* than in *rad9 rad3-E1369K* cells in HU (Fig 3A). In HU-treated *rad3-F2250L* cells with lower Rad3 kinase activity, Rad9 C-terminus significantly increases Mrc1 phosphorylation (Fig 6C). Although the function of the recruited Rad4 in promoting Chk1 to be phosphorylated by Rad3 is relatively clear in fission yeast, whether and how Rad4^TopBP1/Dpb11^ is recruited at the fork for Cds1 phosphorylation remains to be investigated. Surprisingly, in the absence of HU, Rad3-E1369K IPed from *rad9-∆C* showed a significantly higher *in vitro* kinase activity than that from wild-type *rad9* cells (Fig 5 and Fig 6E), suggesting that the Rad9 C-terminus may induce a long-lasting conformational change in Rad3-E1369K to suppress the constitutively active kinase, which may be harmful to the cell under normal conditions. It would be interesting to examine whether the negative regulatory function of the Rad9 C-terminus on Rad3-E1369K is a direct or an indirect effect via other factors. Nevertheless, although further studies are needed, the regulatory effects of the Rad9 C-terminus may play an important role in fine-tuning Rad3 kinase signaling at the fork.

### Different initiation mechanisms of the DRC and the DDC pathways

Although the constitutively active Rad3-E1369K significantly promotes Cds1 phosphorylation in *rad9-∆C*, it does not stimulate Chk1 phosphorylation in the same *rad9* phospho-mutant (Fig 3B), showing that at the DNA damage site, Rad3 kinase signaling is regulated by a different, not a universal, checkpoint initiation mechanism. In support of this notion, we have previously reported that while the cooperation of the RPA-binding domain and the KKRK motif in Rad26 is crucial for the Rad3 signaling at the fork, it is less important at the DNA damage site, particularly at the site of strand breaks [37]. As shown in Fig 1, while Crb2^53 BP1/Rad9^ is required for Chk1 activation, it plays a minimal role in the phosphorylation of Cds1 and Mrc1, suggesting that even in the presence of a constitutively active Rad3, Chk1 has to be properly positioned by the collaboration of the recruited Rad4, Crb2, or other proteins for its phosphorylation. In support of this possibility, previous studies have identified several repair proteins that contribute to checkpoint initiation at the DNA damage site in *Xenopus* and mammalian cells [50–54]. Whether a repair protein modulates the checkpoint sensor kinase activity in a genetically tractable system such as *S. pombe* remains to be investigated.

## Materials and methods

### Yeast strains and plasmids

The *S. pombe* strains used in the study were cultured in YE6S liquid media containing 0.5% yeast extract, 3% dextrose, with six supplements or in EMM6S media. The yeast strains, plasmids, and PCR primers used in the study are listed in S1-S3 Tables. The mutations were identified by whole genome sequencing (Innomics, Inc.) and confirmed by Sanger sequencing (Retrogen).

### Drug sensitivity assays

Sensitivities of wild-type and the mutant *S. pombe* to HU and MMS were measured using the spot assay or colony recovery as previously described [34,55].

### Suppressor screen

*rad9* phospho-mutants were cultured in EMM6S liquid media to log phase, harvested, and washed with 50 mM Tris-Maleate buffer, pH 6.0, and resuspended in the same buffer at a cell density of 35.0 OD/ml. A 250 µl aliquot of the cell suspension was incubated with 0.2 mg/ml MNNG (N-methyl-N′-nitro-N-nitrosoguanidine) at 25°C for 90 min [23]. The mutagenized cells were washed twice and incubated in 5 mL of EMM6S medium at 25°C for 3 h to recover. The cells were then spread on YE6S media containing 5 mM HU and phloxine B. The plates were incubated at 30°C for 4–5 days to allow colony formation.

### AlphaFold2 modeling

The protein sequences of *S. pombe* Rad3, Rad3-E1369K, and Rad26 were submitted to the AlphaFold2 server (https://*alphafoldserver*.com/). The predicted models of Rad3-Rad26 and Rad3-E1369K-Rad26 were analyzed in PyMOL.

### IP and co-IP

5 OD logarithmically growing cells were harvested and lysed using mini-bead beater in a buffer containing 25 mM HEPES/NaOH (pH 7.5), 50 mM NaF, 1 mM NaVO_4_, 10 mM NaP_2_O_7_, 40 mM ß-glycerophosphate, 0.1% Tween 20, 0.5% NP-40, and protease inhibitors. The lysates were centrifuged at 16,000 g at 4˚C for 10 min. The resulting supernatant was used as a whole-cell extract. Agarose resin cross-linked to anti-HA or anti-myc antibodies was washed three times with Tris-buffered saline containing 0.05% Tween 20 (TBS-T) before being incubated with the whole-cell extract by rotating at 4˚C for 2 h. The samples were washed three times with TBS-T at 4˚C for 10 min. The IP samples were separated by 8% SDS-PAGE followed by Western blotting.

### Western blotting

Rad3-dependent phosphorylation of Cds1-T11 and Mrc1-T645 was analyzed by Western blotting using the phospho-specific antibodies described in previous studies [18]. Mrc1 was detected by a polyclonal antibody on the same membrane [55]. Rad3 was tagged with a 10xMyc epitope at the N-terminus, while Cds1 and Rad26 were tagged with an HA epitope at the C- and N-terminus, respectively, and detected by Western blotting using anti-myc or anti-HA antibodies. Phosphorylation of Chk1 by Rad3 was examined by a mobility shift assay [21]. The large subunit of RPA Ssb1 was detected using an anti-Ssb1 antibody [55]. The blotting signal was detected by electrochemiluminescence using the ChemiDoc XRS Imaging system (BioRad). Signal intensities of the bands were quantified and analyzed by ImageLab (BioRad).

### *In vitro* Rad3 kinase assay

Whole cell extracts were prepared as described above for IP, except that 15 OD cells were harvested. Pre-washed Protein G Dynabeads were incubated with anti-myc antibodies at 25°C for 60 min. The whole-cell extracts were then incubated with the antibodies bound to Dynabeads by rotating at 4˚C for 5 h. After a quick wash in TBS-T containing protease inhibitors, the beads were collected in a magnetic rack, and the IPed Rad3 was examined by the kinase assay using bacterially purified kinase-inactive Cds1(D312E) as the substrate [19,26]. Briefly, a 20 µl kinase reaction mixture of 50 mM Tris-HCl, pH 7.5, 10 mM MgCl_2_, 2 mM DTT, 0.1 mM EDTA, 0.01% NP-40, 200 µM ATP, and 0.48 µM of Cds1(D312E) was added to the beads. The reactions were incubated at 25°C for 60 min and then stopped by adding SDS gel loading buffer. After heating at 92˚C for 10 min, the sample was analyzed by SDS PAGE. After transferring to a nitrocellulose membrane, Cds1(D312E) was revealed by Ponceau S staining followed by Western blotting using phosphor-specific antibody to detect Cds1-pT11. The upper part of the membrane was used to detect Rad3 or Rad26 using anti-myc or anti-HA antibody, respectively.

### DNA pull-down assay

The assay was conducted using the previously described method [38]. Briefly, whole-cell extract was prepared from 15 OD cells using a mini-bead beater and acid-washed glass beads in 2X buffer containing 100 mM HEPES, pH 7.4, 400 mM NaOAc, 100 mM MgOAc, 1 mM EDTA, 1 mM sodium orthovanadate, 0.1% 2-mercaptoethanol, 1% (w/v) Triton X-100, and protease inhibitors. The lysates were clarified at 16,000g, 4˚C, for 5 min. A 72 base pair dsDNA (10 µg) created by annealing P1-Biotin/P2 oligonucleotides was incubated with 4 mg streptavidin-coated magnetic Dynabeads pre-equilibrated in 50 mM HEPES, pH 7.4, containing 1 M NaCl, washed 4x with 50 mM HEPES, pH 7.4, containing 0.15 M NaCl, and finally resuspended in the same buffer at 25°C for 30 min in 400 µl. 10 µl of this suspension was incubated with the whole cell extract at 4°C for 60 min on a shaking platform. The beads were collected in a magnetic rack and washed three times in buffer containing 50 mM HEPES (pH 7.4), 0.1 M NaCl, 1% Triton X-100, 0.1% 2-mercaptothanol, and protease inhibitors. The beads were resuspended in SDS-loading buffer and subjected to 8% SDS-PAGE followed by Western blotting to detect Rad26 and Rad3.

## Supporting information

S1 Fig Schematic for the screening of suppressors of *rad9* phospho-mutants. The HU-sensitive *rad9-T412A* and *rad9-ΔC* strains were exposed to the mutagen MNNG (N-methyl-N′-nitro-N-nitrosoguanidine) to achieve approximately 90% killing [23]. The cells were allowed to recover in rich medium for 2–3 h before spreading onto plates containing 5 mM HU and phloxine B, a lethality dye. The HU-resistant colonies were selected, streaked out into single colonies, and tested by spot assays for HU resistance. After backcrossing with the parental *rad9-T412A* or *rad9-ΔC*, the suppressors were examined for Cds1 phosphorylation, which identified the *L11* suppressor that restored Cds1 phosphorylation and HU-resistance in both *rad9-T412A* and *rad9-∆C* mutants. After backcrossing *L11* with *rad9-∆C*, the HU-resistant colonies with restored Cds1 phosphorylation were pooled for purification of genomic DNA and subsequent genome sequencing. The HU-sensitive colonies were similarly pooled for genome sequencing as the reference. The *rad3-E1369K* mutation identified in *L11* by the genome sequencing was then confirmed by Sanger sequencing.
(TIF)

S2 Fig Screening of the *L11* suppressor of *rad9-T412A* with increased Rad3 phosphorylation of Mrc1 and Cds1. (A) Drug sensitivities of the screened suppressors of the K series isolated in *rad9-∆C* and the L series in *rad9-T412A* were determined by spot assay. Wild-type, *rad3∆, rad9-ΔC*, and *rad9-T412A* strains were included as controls. (B) Phosphorylation of Cds1 in the screened suppressors was examined by Western blotting using the phospho-specific antibody against Cds1-pT11. Among the suppressors, only L11 rescued the Cds1 phosphorylation in *rad9-T412A* or *rad9-∆C*. (C) Chk1 phosphorylation was examined by mobility shift assay. Wild-type *S. pombe*, *rad9-T412A*, and the *L11* suppressor were treated with (+) or without (-) 0.01% MMS for 90 min. Whole-cell lysates were analysed by SDS-PAGE followed by Western blotting with anti-HA antibodies to detect the C-terminally tagged Chk1 (top panel). A section of the Ponceau S-stained membrane is shown as the loading control. The upper-shifted phosphorylation band was quantified and shown at the bottom as the ratio of phosphorylated Chk1 vs total Chk1. (D) Mrc1 phosphorylation in the *L11* suppressor was examined by Western blotting using the phospho-specific antibody against Mrc1-pT645 as in Fig 1C.
(TIF)

S3 Fig The *L11* suppressor also rescues *rad9-∆C.* (A) The *L11* suppressor rescues both *rad9-T412A* and *rad9-∆C* in HU and MMS. *L11* was crossed into *rad9-ΔC,* and the drug sensitivities were determined by spot assay, in which a series of 5-fold dilutions of the cells was spotted on plates containing HU or MMS at the indicated concentrations. (B) The *L11 rad9-∆C* cells were transformed with plasmids expressing Rad4 (also known as Cut5), Suc22, the small subunit of ribonucleotide reductase, Cds1, and Rad9 under their native promoters. The drug sensitivities were examined by the three-spot assay as in (A), except the cells were diluted in a 10-fold series.
(TIF)

S4 Fig Alignment of fission yeast Rad3, budding yeast Mec1, and human ATR, and the locations of the *E1369K* and *F2250L* mutations in Rad3. (A) The primary amino acid sequences of *S. pombe* Rad3, *S. cerevisiae* Mec1, and human ATR were aligned by CLUSTALW using MacVector. The less conserved N-terminal HEAT repeat region is not shown. The N-FAT, M-FAT, C-FAT, kinase domain, PRD, and FATC are highlighted by blue, green, purple, and brown lines, respectively [16]. The activation loop, the KαC helix, and the PRD-I in the PRD of the kinase domain are marked by red squares and arrows, respectively. The E1369K mutation in N-FAT and the F2250L mutation in the activation loop of Rad3 are marked in red. (B) Y1623 in ATR, the equivalent residue of Rad3-E1369, is located close to the catalytic centre but distant from the PRD in the cryo-EM structure of ATR-ATRIP [32]. Also highlighted in the ATR-ATRIP structure are the activation loop, PRD-I, and the KαC helix.
(TIF)

S5 Fig Extrachromosomal expression of Rad3-E1369K rescues the drug sensitivities of *rad9-∆C* by promoting Rad3 phosphorylation of Cds1 and Mrc1. (A) The drug sensitivities of the double mutant *rad9-ΔC Δrad3* expressing wild-type Rad3 or Rad3-E1369K on a vector under the control of the *rad3* promoter were examined by spot assay. The double mutant with an empty vector was used as a control. (B) Western blotting confirms similar expression levels of Rad3 and Rad3-E1369K using the anti-myc antibody to detect the N-terminal epitope tag. Asterisk indicates a cross-reacting material. (C) Cds1 phosphorylation was examined in the strains used in (A) by Western blotting before (-) or after (+) HU treatment. (D) Mrc1 phosphorylation was examined by Western blotting in the strains used in (A).
(TIF)

S6 Fig The primary *L11* mutant carries a secondary uncharacterized mutation that promotes cell survival in oxidative stress caused by chronic HU exposure. (A) A schematic of the integration of the *rad3-E1369K* mutation at the genomic locus. *rad3* was tagged with a 10myc epitope at the N-terminus and the *nmt1* terminator (nmtT) to replace its own terminator, followed by a *kanR* marker. The DNA fragment was released from the vector by digestion with SalI and SbfI, gel-purified, and transformed into wild-type *S. pombe*. G418-resistant colonies were screened by colony PCR and subsequent Western blotting to detect the N-terminal myc tag. The integrated mutation was confirmed by Sanger sequencing. (B) Drug sensitivities of the primary *L11* suppressor carrying the *rad9-T412A* or *rad9-ΔC* mutation were compared with the *rad3-E1369K* integrant carrying the *rad9-∆C* mutation. (C) Tetrad dissection analysis of the crosses between *L11 rad9-T412A* and *rad9-ΔC*. Colonies formed on a YE6S plate were replicated onto a YE6S plate lacking adenine to show a 2:2 ratio of the two *ade6* alleles, which confirmed the dissection. The colonies were also replicated onto a YE6S plate containing 5 mM HU and phloxine B. Tetrads with three HU-resistant spores were marked by green dashed squares. Dashed lines indicate discontinuity. (D) Antioxidant N-acetyl cysteine significantly increased the HU-resistance in *rad3-E1369K* integrant carrying the *rad9-∆C* mutation.
(TIF)

S7 Fig The *rad3-E1369K* mutation does not rescue *rad9∆* and *rad1∆* mutants of the 9-1-1 complex, and the 9-1-1 loader *rad17∆* mutant in HU. The integrated *rad3-E1369K* mutation was crossed into *rad9∆, rad1∆,* and *rad17∆* strains. Four individual colonies from each cross were selected for the HU sensitivity assay. Wild-type, *rad3∆*, and the parental strains were used as the controls. Rad9 and Rad1 bind to Hus1 to form the 9-1-1 clamp complex, while Rad17 is the loader of the 9-1-1 complex.
(TIF)

S8 Fig The charge reversal *E1369K* mutation does not affect Rad3 binding to Rad26 and the recruitment of the Rad3-Rad26 complex to DNA. (A) AlphaFold2 structures of the Rad3-Rad26 heterodimer containing Rad3 (left) or Rad3-E1369K (right). Rad3 is shown in green, while Rad26 is in orange. E1369 resides in a SRESS motif, forming a small loop connecting the α-helices. The mutation alters the local structure, enabling binding of the N-terminus of Rad26 to the Rad3 kinase domain. (B) The E1369K mutation did not affect the interactions between Rad3 and Rad26, and Rad3-Rad26 with Ssb1, the large subunit of RPA. Rad26 was IPed using an anti-HA antibody from whole-cell extracts expressing Rad26 (top panel on the right). The co-IPed Rad3 and Ssb1 were analysed by Western blotting using anti-myc and anti-Ssb1 antibodies, respectively (middle and bottom panels on the right). A 2% portion of the whole cell extract was analysed as input in the left three panels. Asterisk indicates a non-specific band. (C) DNA pull-down assay for Rad3 and Rad3-E1369K in complex with wild-type Rad26 or Rad26 with the indicated mutations. Whole cell extracts were incubated with a 72 bp dsDNA bound to magnetic beads. The beads were washed three times and analysed by Western blotting using anti-HA antibody to detect Rad26 (top panel) and anti-myc antibody to detect Rad3 (middle panel). A portion of the Ponceau S-stained membrane is shown for the loading (bottom).
(TIF)

S9 Fig The IPed Rad3-E1369K showed a constitutively increased kinase activity *in vitro* regardless of the Rad26 N-terminal mutations. Rad3 and Rad3-E1369K were IPed from the *rad26Δ rad9-ΔC* double mutant expressing Rad26 or Rad26 mutants with the indicated mutations. The *in vitro* Rad3 kinase assay was conducted using the kinase-dead Cds1(D312E) substrate as described in Materials and Methods. Phosphorylated Cds1 was detected using a phospho-specific antibody against Cds1-pT11, quantified, and the results are shown at the bottom in percentages. A portion of the Ponceau S-stained membrane containing Cds1 substrate and the IgG used for IP is shown in the bottom panel.
(TIF)

S1 Table List of *S. pombe* strains used in this study. (PDF)

S2 Table List of plasmids used in this study. (PDF)

S3 Table List of primers used in this study. (PDF)
