# Supplementary figures and images for: A point mutation in the FAT domain constitutively increases the kinase activity of Rad3ATR and bypasses the requirement for 9-1–1 phosphorylation to activate the DNA replication checkpoint
Source: PLoS Genet. 2026 Jun 22;22(6):e1012213. doi: 10.1371/journal.pgen.1012213 (PMC13309046; doi:10.1371/journal.pgen.1012213)

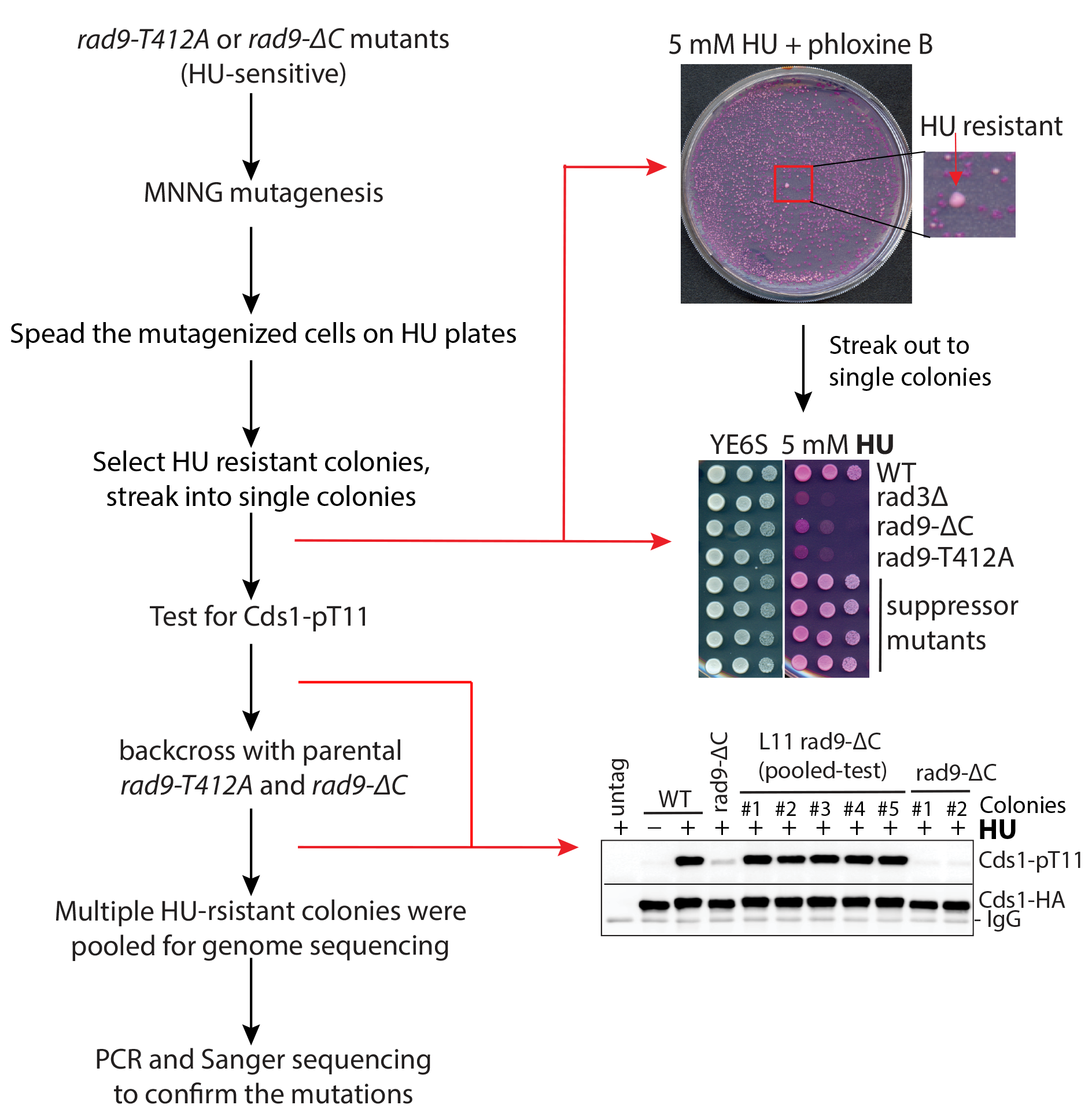

Supplement: S1 Fig — The HU-sensitive rad9-T412A and rad9-ΔC strains were exposed to the mutagen MNNG (N-methyl-N′-nitro-N-nitrosoguanidine) to achieve approximately 90% killing [23]. The cells were allowed to recover in rich medium for 2–3 h before spreading onto plates containing 5 mM HU and phloxine B, a lethality dye. The HU-resistant colonies were selected, streaked out into single colonies, and tested by spot assays for HU resistance. After backcrossing with the parental rad9-T412A or rad9-ΔC, the suppressors were examined for Cds1 phosphorylation, which identified the L11 suppressor that restored Cds1 phosphorylation and HU-resistance in both rad9-T412A and rad9-∆C mutants. After backcrossing L11 with rad9-∆C, the HU-resistant colonies with restored Cds1 phosphorylation were pooled for purification of genomic DNA and subsequent genome sequencing. The HU-sensitive colonies were similarly pooled for genome sequencing as the reference. The rad3-E1369K mutation identified in L11 by the genome sequencing was then confirmed by Sanger sequencing. (TIF) [file pgen.1012213.s001.tif]

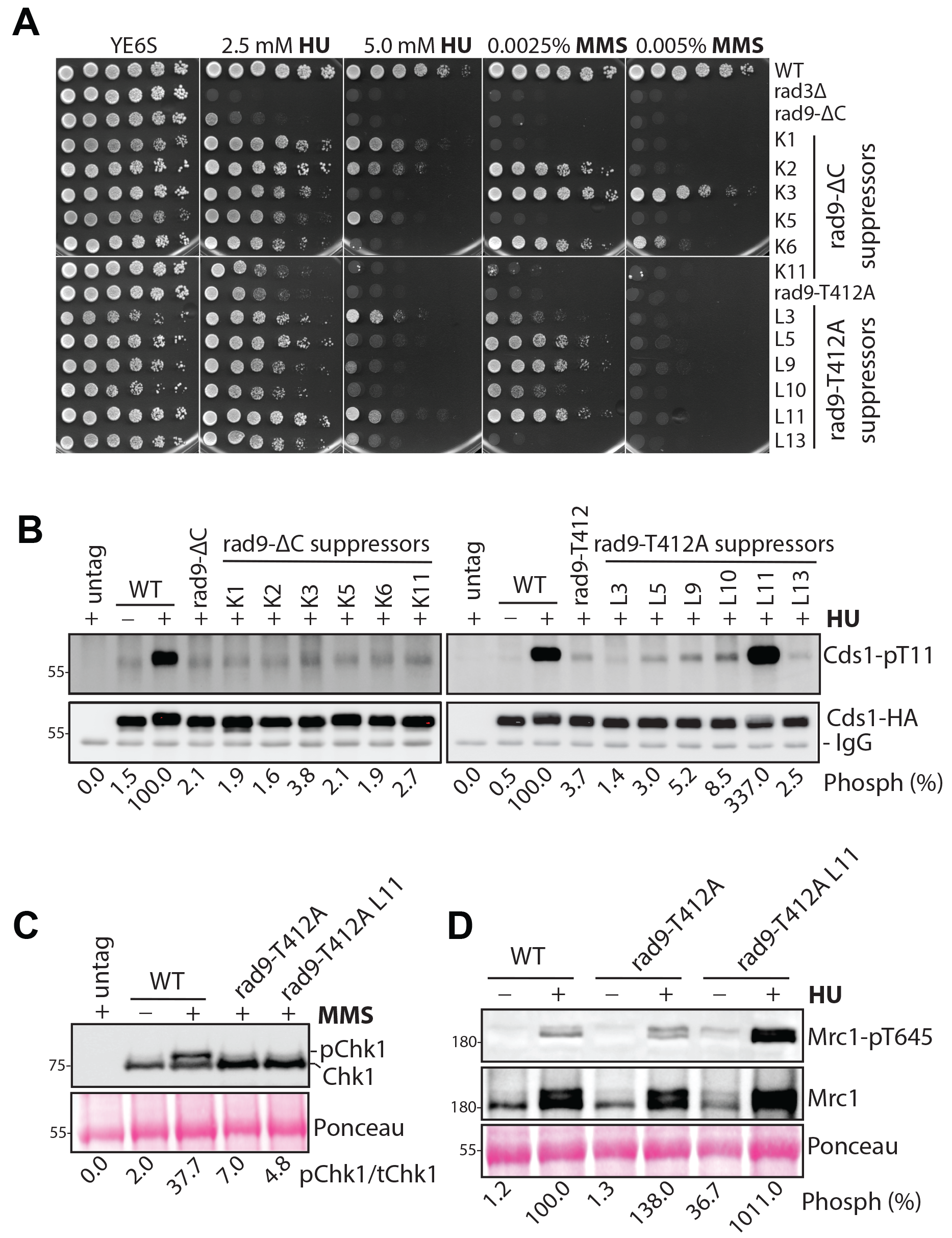

Supplement: S2 Fig — (A) Drug sensitivities of the screened suppressors of the K series isolated in rad9-∆C and the L series in rad9-T412A were determined by spot assay. Wild-type, rad3∆, rad9-ΔC, and rad9-T412A strains were included as controls. (B) Phosphorylation of Cds1 in the screened suppressors was examined by Western blotting using the phospho-specific antibody against Cds1-pT11. Among the suppressors, only L11 rescued the Cds1 phosphorylation in rad9-T412A or rad9-∆C. (C) Chk1 phosphorylation was examined by mobility shift assay. Wild-type S. pombe, rad9-T412A, and the L11 suppressor were treated with (+) or without (-) 0.01% MMS for 90 min. Whole-cell lysates were analysed by SDS-PAGE followed by Western blotting with anti-HA antibodies to detect the C-terminally tagged Chk1 (top panel). A section of the Ponceau S-stained membrane is shown as the loading control. The upper-shifted phosphorylation band was quantified and shown at the bottom as the ratio of phosphorylated Chk1 vs total Chk1. (D) Mrc1 phosphorylation in the L11 suppressor was examined by Western blotting using the phospho-specific antibody against Mrc1-pT645 as in Fig 1C. (TIF) [file pgen.1012213.s002.tif]

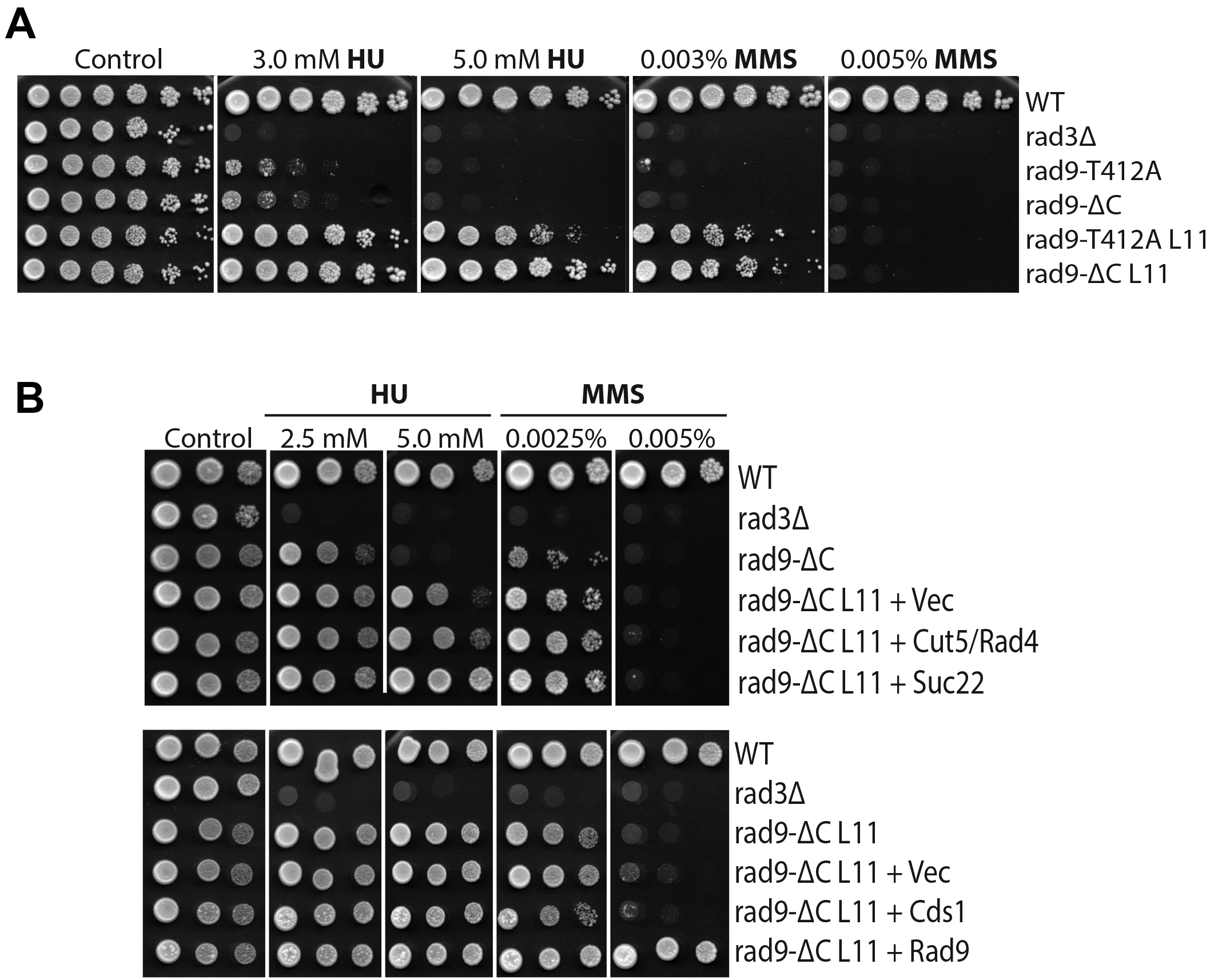

Supplement: S3 Fig — (A) The L11 suppressor rescues both rad9-T412A and rad9-∆C in HU and MMS. L11 was crossed into rad9-ΔC, and the drug sensitivities were determined by spot assay, in which a series of 5-fold dilutions of the cells was spotted on plates containing HU or MMS at the indicated concentrations. (B) The L11 rad9-∆C cells were transformed with plasmids expressing Rad4 (also known as Cut5), Suc22, the small subunit of ribonucleotide reductase, Cds1, and Rad9 under their native promoters. The drug sensitivities were examined by the three-spot assay as in (A), except the cells were diluted in a 10-fold series. (TIF) [file pgen.1012213.s003.tif]

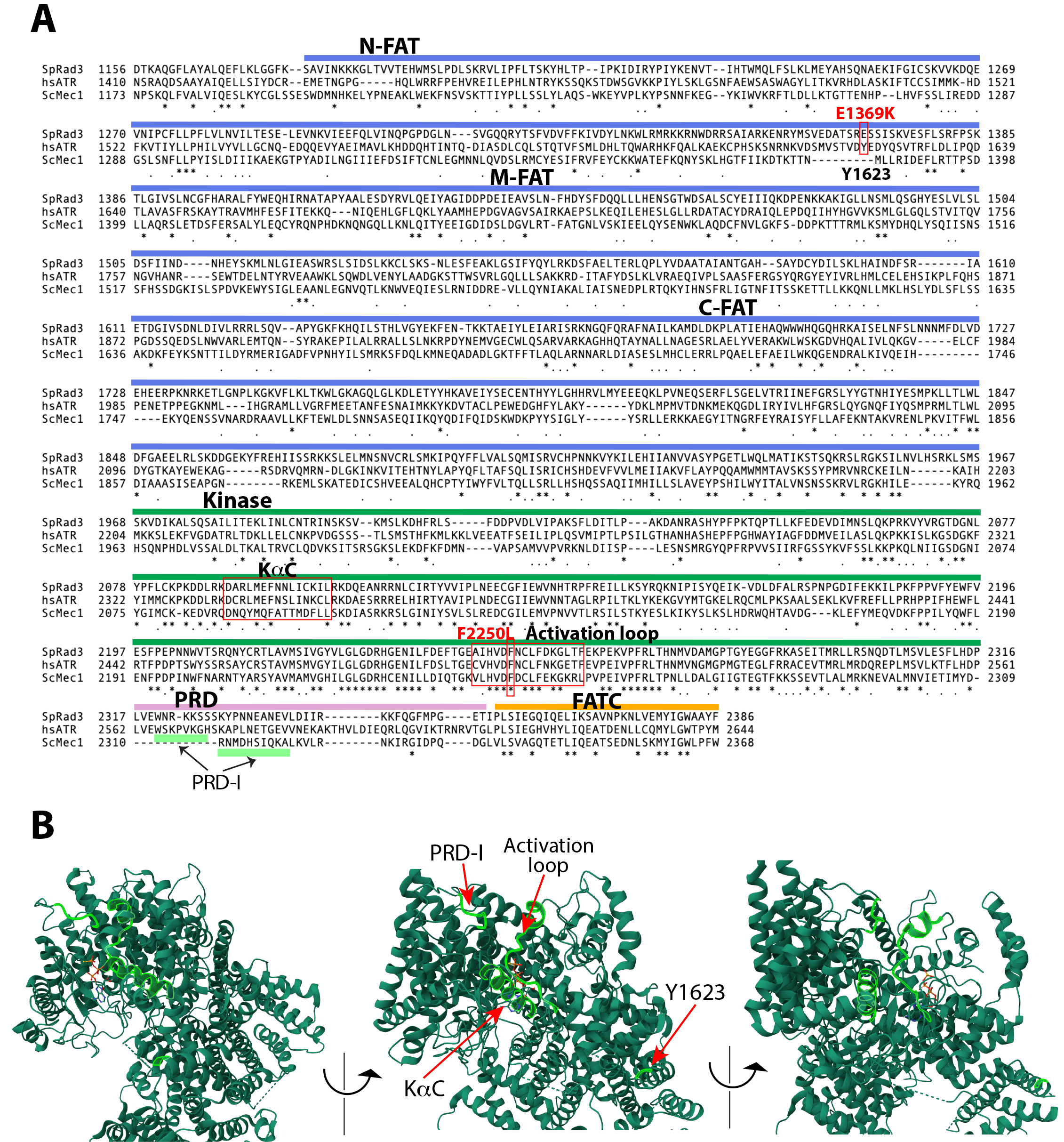

Supplement: S4 Fig — (A) The primary amino acid sequences of S. pombe Rad3, S. cerevisiae Mec1, and human ATR were aligned by CLUSTALW using MacVector. The less conserved N-terminal HEAT repeat region is not shown. The N-FAT, M-FAT, C-FAT, kinase domain, PRD, and FATC are highlighted by blue, green, purple, and brown lines, respectively [16]. The activation loop, the KαC helix, and the PRD-I in the PRD of the kinase domain are marked by red squares and arrows, respectively. The E1369K mutation in N-FAT and the F2250L mutation in the activation loop of Rad3 are marked in red. (B) Y1623 in ATR, the equivalent residue of Rad3-E1369, is located close to the catalytic centre but distant from the PRD in the cryo-EM structure of ATR-ATRIP [32]. Also highlighted in the ATR-ATRIP structure are the activation loop, PRD-I, and the KαC helix. (TIF) [file pgen.1012213.s004.tif]

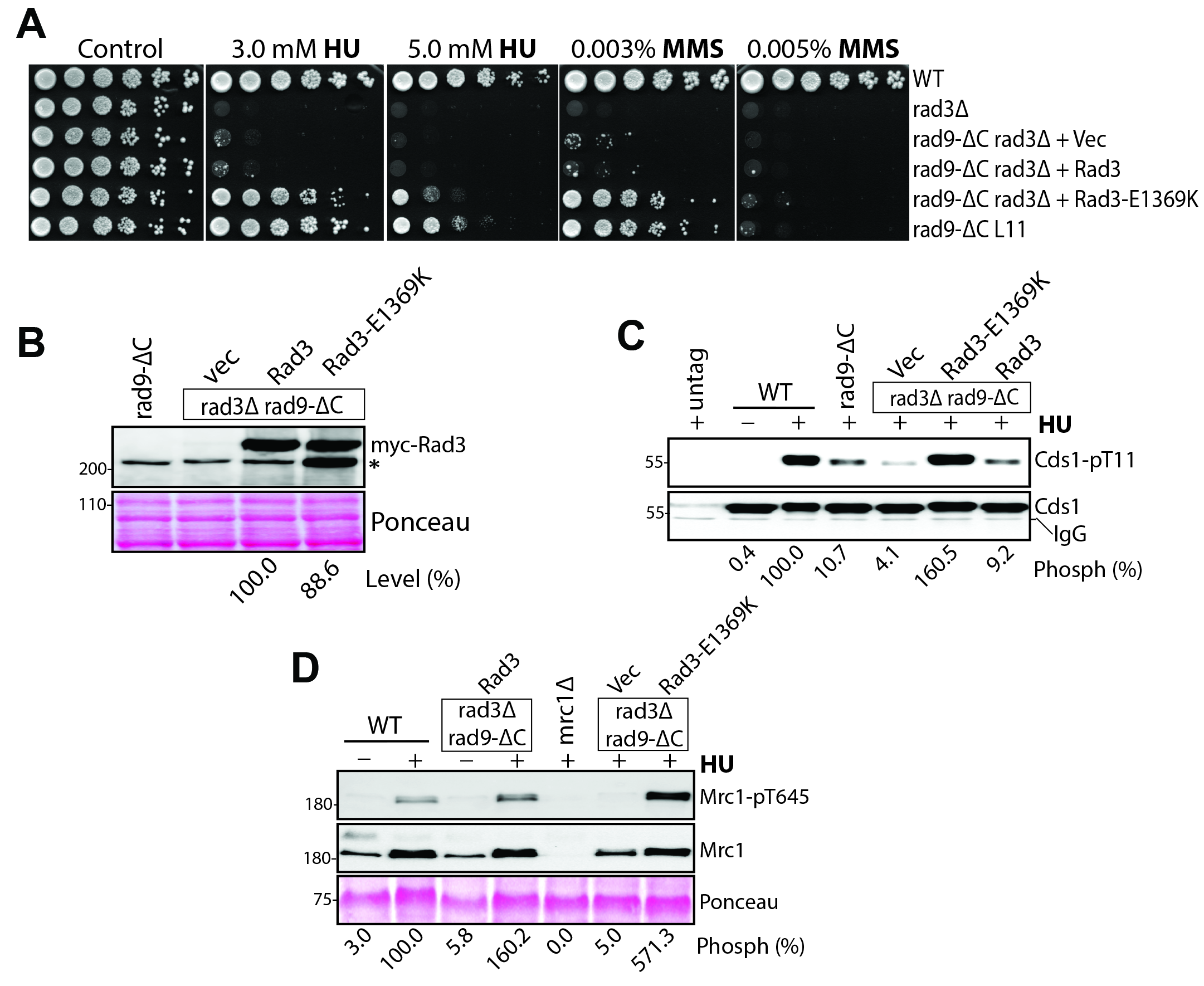

Supplement: S5 Fig — (A) The drug sensitivities of the double mutant rad9-ΔC Δrad3 expressing wild-type Rad3 or Rad3-E1369K on a vector under the control of the rad3 promoter were examined by spot assay. The double mutant with an empty vector was used as a control. (B) Western blotting confirms similar expression levels of Rad3 and Rad3-E1369K using the anti-myc antibody to detect the N-terminal epitope tag. Asterisk indicates a cross-reacting material. (C) Cds1 phosphorylation was examined in the strains used in (A) by Western blotting before (-) or after (+) HU treatment. (D) Mrc1 phosphorylation was examined by Western blotting in the strains used in (A). (TIF) [file pgen.1012213.s005.tif]

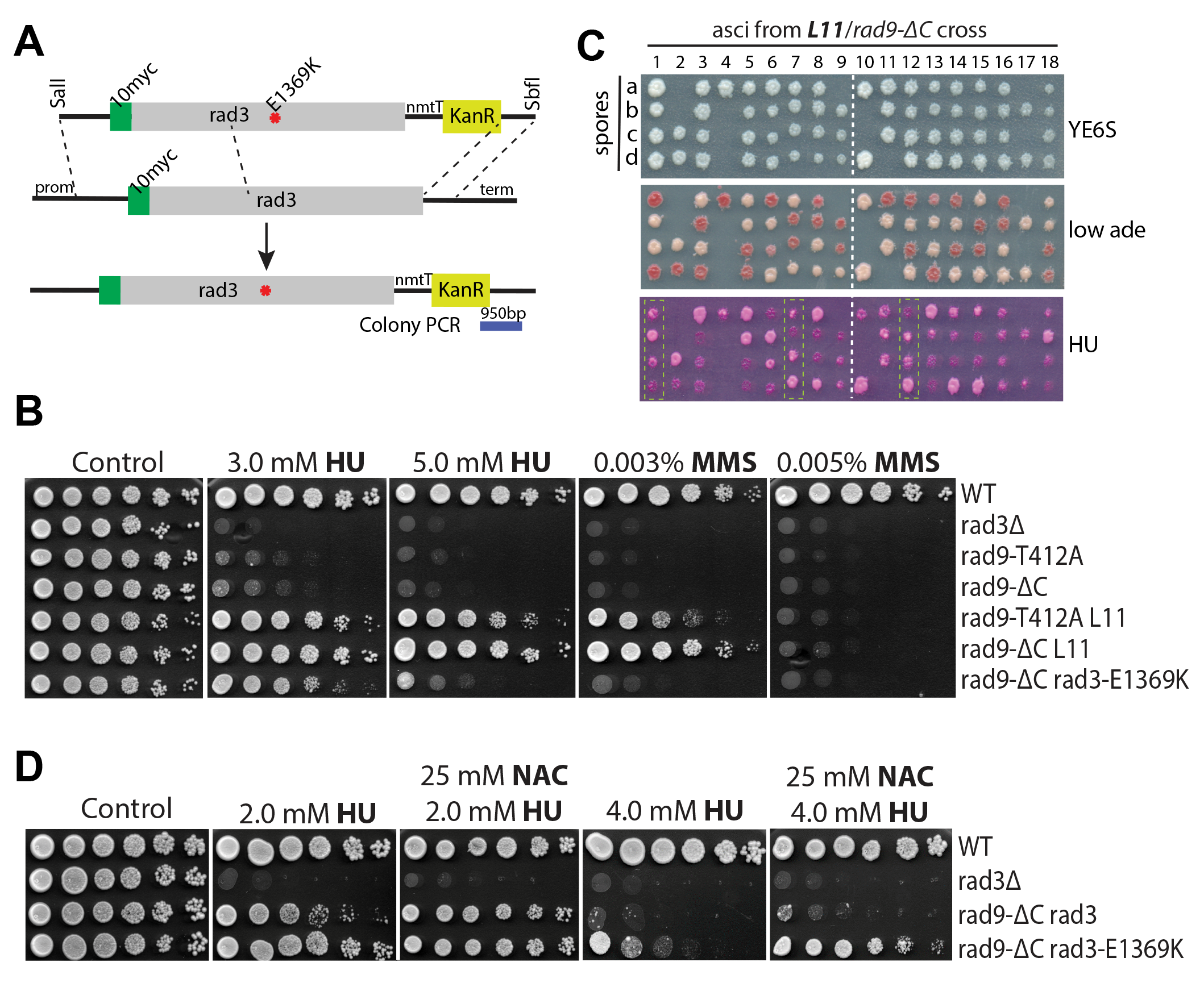

Supplement: S6 Fig — (A) A schematic of the integration of the rad3-E1369K mutation at the genomic locus. rad3 was tagged with a 10myc epitope at the N-terminus and the nmt1 terminator (nmtT) to replace its own terminator, followed by a kanR marker. The DNA fragment was released from the vector by digestion with SalI and SbfI, gel-purified, and transformed into wild-type S. pombe. G418-resistant colonies were screened by colony PCR and subsequent Western blotting to detect the N-terminal myc tag. The integrated mutation was confirmed by Sanger sequencing. (B) Drug sensitivities of the primary L11 suppressor carrying the rad9-T412A or rad9-ΔC mutation were compared with the rad3-E1369K integrant carrying the rad9-∆C mutation. (C) Tetrad dissection analysis of the crosses between L11 rad9-T412A and rad9-ΔC. Colonies formed on a YE6S plate were replicated onto a YE6S plate lacking adenine to show a 2:2 ratio of the two ade6 alleles, which confirmed the dissection. The colonies were also replicated onto a YE6S plate containing 5 mM HU and phloxine B. Tetrads with three HU-resistant spores were marked by green dashed squares. Dashed lines indicate discontinuity. (D) Antioxidant N-acetyl cysteine significantly increased the HU-resistance in rad3-E1369K integrant carrying the rad9-∆C mutation. (TIF) [file pgen.1012213.s006.tif]

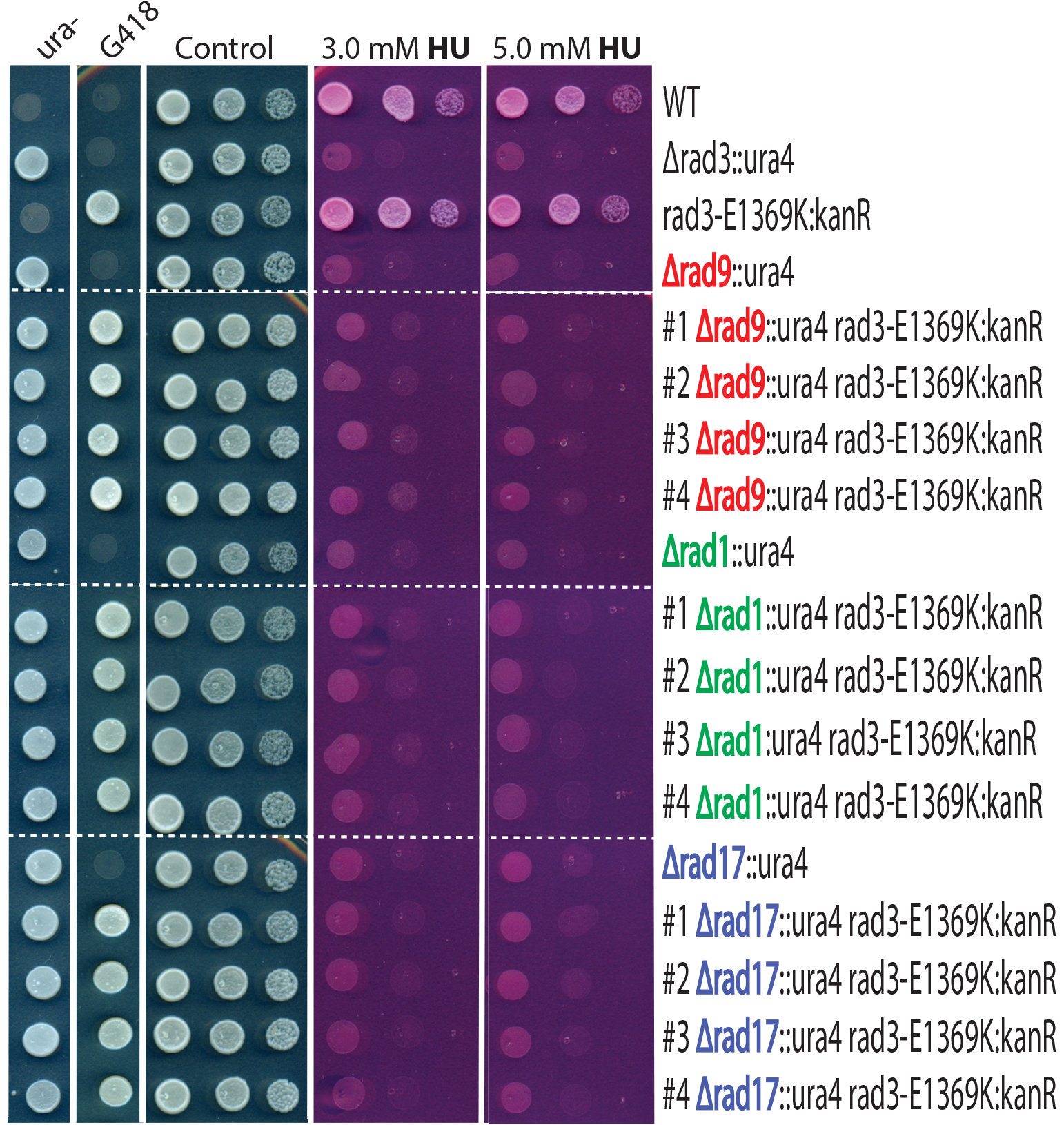

Supplement: S7 Fig — The integrated rad3-E1369K mutation was crossed into rad9∆, rad1∆, and rad17∆ strains. Four individual colonies from each cross were selected for the HU sensitivity assay. Wild-type, rad3∆, and the parental strains were used as the controls. Rad9 and Rad1 bind to Hus1 to form the 9-1-1 clamp complex, while Rad17 is the loader of the 9-1-1 complex. (TIF) [file pgen.1012213.s007.tif]

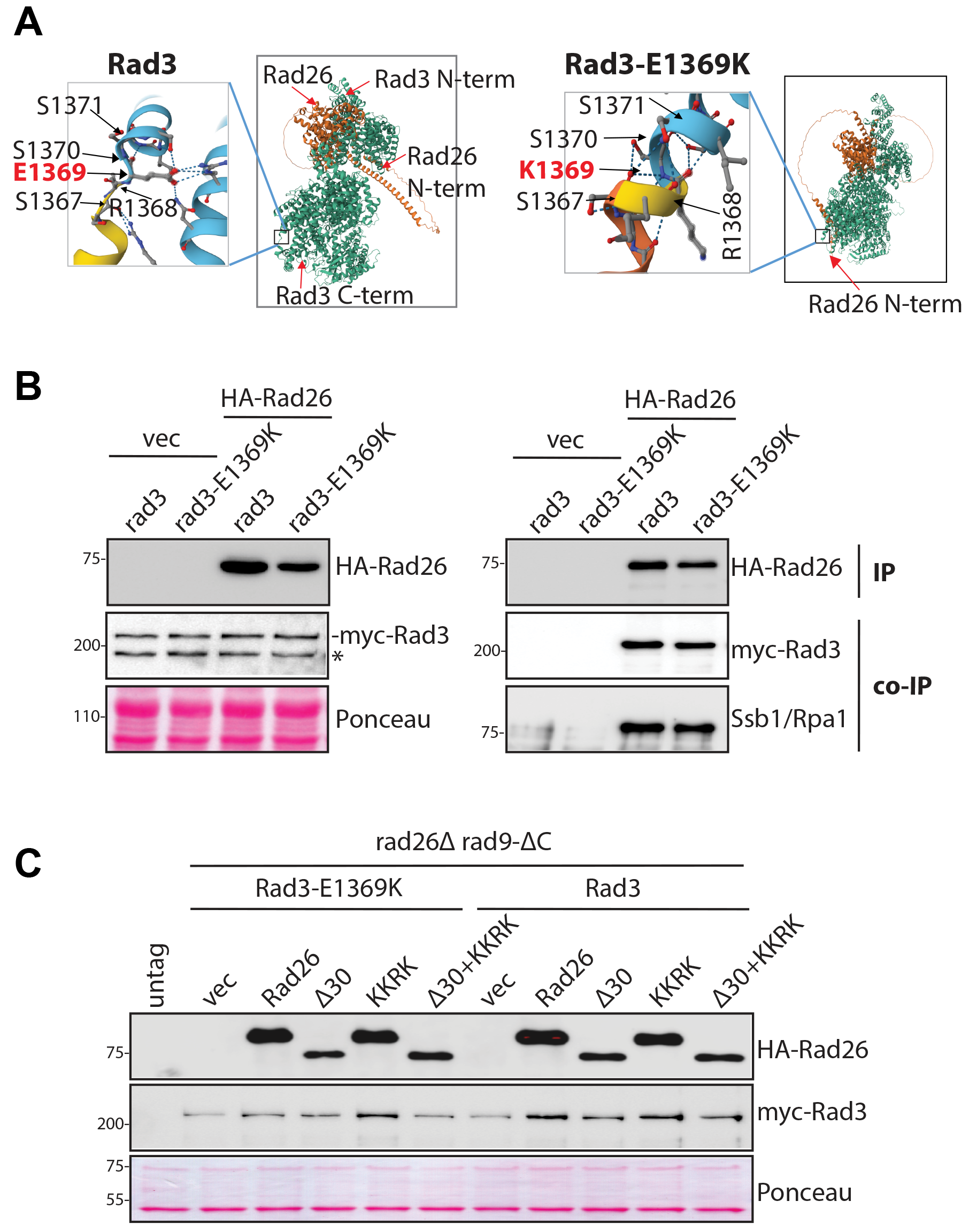

Supplement: S8 Fig — (A) AlphaFold2 structures of the Rad3-Rad26 heterodimer containing Rad3 (left) or Rad3-E1369K (right). Rad3 is shown in green, while Rad26 is in orange. E1369 resides in a SRESS motif, forming a small loop connecting the α-helices. The mutation alters the local structure, enabling binding of the N-terminus of Rad26 to the Rad3 kinase domain. (B) The E1369K mutation did not affect the interactions between Rad3 and Rad26, and Rad3-Rad26 with Ssb1, the large subunit of RPA. Rad26 was IPed using an anti-HA antibody from whole-cell extracts expressing Rad26 (top panel on the right). The co-IPed Rad3 and Ssb1 were analysed by Western blotting using anti-myc and anti-Ssb1 antibodies, respectively (middle and bottom panels on the right). A 2% portion of the whole cell extract was analysed as input in the left three panels. Asterisk indicates a non-specific band. (C) DNA pull-down assay for Rad3 and Rad3-E1369K in complex with wild-type Rad26 or Rad26 with the indicated mutations. Whole cell extracts were incubated with a 72 bp dsDNA bound to magnetic beads. The beads were washed three times and analysed by Western blotting using anti-HA antibody to detect Rad26 (top panel) and anti-myc antibody to detect Rad3 (middle panel). A portion of the Ponceau S-stained membrane is shown for the loading (bottom). (TIF) [file pgen.1012213.s008.tif]

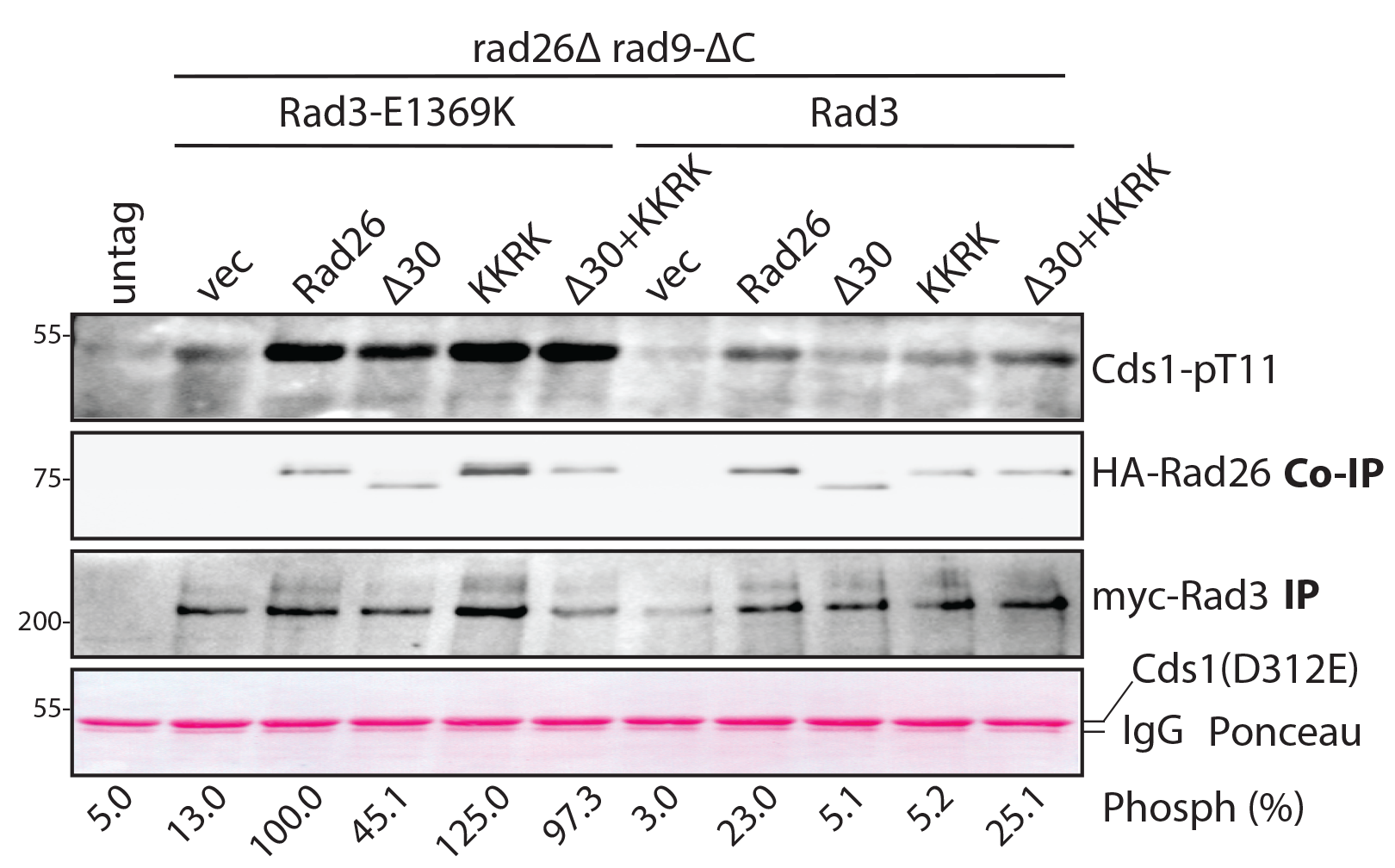

Supplement: S9 Fig — Rad3 and Rad3-E1369K were IPed from the rad26Δ rad9-ΔC double mutant expressing Rad26 or Rad26 mutants with the indicated mutations. The in vitro Rad3 kinase assay was conducted using the kinase-dead Cds1(D312E) substrate as described in Materials and Methods. Phosphorylated Cds1 was detected using a phospho-specific antibody against Cds1-pT11, quantified, and the results are shown at the bottom in percentages. A portion of the Ponceau S-stained membrane containing Cds1 substrate and the IgG used for IP is shown in the bottom panel. (TIF) [file pgen.1012213.s009.tif]
