# Supplementary material for: A point mutation in the FAT domain constitutively increases the kinase activity of Rad3ATR and bypasses the requirement for 9-1–1 phosphorylation to activate the DNA replication checkpoint
Source: PLoS Genet. 2026 Jun 22;22(6):e1012213. doi: 10.1371/journal.pgen.1012213 (PMC13309046; doi:10.1371/journal.pgen.1012213)
Supplement: S1 Table — (PDF) [file pgen.1012213.s010.pdf]

S1 Table. List of *S. pombe* strains used in this study.

Strain	Genotype	Sources
TK7	*h- leu1-32 ura4-D18 ade6-M210*	Lab stock
NR1826	*h⁻ rad3::ura4 leu1-32 ura4-D18 ade6*	Lab stock
LLD3427	*h- chk1-9myc2HA6his:ura4 leu1-32 ura4-D18*	Lab stock
YJ1139	*h- 10myc-rad3(D2249E)*	Lab stock
YJ418	*h+ Δmrc1::ura4 cds1-6his2HA*	Lab stock
YJ911	*h?Δmrc1::ura4 leu1:mrc1(T645A+T653A)3HA cds1-6his2HA*	Lab stock
YJ2630	*h- 10myc-rad3(F2250L)-nmtT:kanR*	Lab stock
YJ1138	*h- 10myc-rad3*	Lab stock
YJ871	*h+ Δrad9::ura4 cds1-6his2HA*	Lab stock
YJ909	*h+Δrad1::ura4+ cds1-6his2HA(int)*	Lab stock
YJ914	*h+Δrad17::ura4+ cds1-6his2HA(int)*	Lab stock
YJ1843	*h+ rad9(wt cDNA int) cds1-6hisHA leu1-32 ura4-D18*	Lab stock
YJ1844	*h+ rad9 (T412A cDNA int) cds1-6hisHA leu1-32 ura4-D18*	Lab stock
YJ1846	*h+ rad9 (Δ411-426 cDNA int) cds1-6hisHA leu1-32 ura4-D18*	Lab stock
YJ906	*h+ Δcrb2::ura4 cds1-6his2HA leu1-32 ura4-D18*	Lab stock
YM424	*cut5 (Δ498-648) nmtT (Leu2) cut5-C term*	Lab stock
KD2229	*h+ rad9 (T412A cDNA int) L11 suppressor cds1-6hisHA leu1-32 ura4-D18*	This study
KD2376	*h- rad9 (Δ411-426 cDNA int) L11 suppressor cds1-6hisHA leu1-32 ura4-D18*	This study
KD2239	*h+ rad9 (Δ411-426 cDNA int) L11 suppressor cds1-6hisHA leu1-32 ura4-D18 [pIRT-2L]*	This study
KD2240	*h+ rad9 (Δ411-426 cDNA int) L11 suppressor cds1-6hisHA leu1-32 ura4-D18 prom Cut5(BamHI-XmaI)nmt Term [pIRT-2U]*	This study
KD2241	*h+ rad9 (Δ411-426 cDNA int) L11 suppressor cds1-6hisHA leu1-32 ura4-D18 prom Suc22+term (BamHI-SalI) [pIRT-2U]*	This study
KD2209	*h+ rad9 (Δ411-426 cDNA int) L11 suppressor cds1-6hisHA leu1-32 ura4-D18 prom-Rad9(SalI-XmaI) [pIRT-2L]*	This study
KD2409	*h+ Δrad3::ura4 rad9(Δ411-426 cDNA int) cds1-6his2HA*	This study
KD2861	*h+Δrad3::ura4 rad9(Δ411-426 cDNA int) cds1-6his2HA [pIRT-2L]*	This study
KD2862	*h+Δrad3::ura4 rad9(Δ411-426 cDNA int) cds1-6his2HA prom-10myc-Rad3/LEU2*	This study
KD2863	*h+Δrad3::ura4 rad9(Δ411-426 cDNA int) cds1-6his2HA prom-10myc-rad3(E1369K)/LEU2*	This study
KD2270	*h+ rad9 (T412A cDNA int) cds1-6hisHA chk1-9myc2HA6his:ura4 leu1-32 ura4-D18*	This study
KD2271	*h+ rad9 (Δ411-426 cDNA int) cds1-6hisHA chk1-9myc2HA6his:ura4 leu1-32 ura4-D18*	This study
KD2272	*h? rad9 (T412A cDNA int) L11 suppressor cds1-6hisHA chk1-9myc2HA6his:ura4 leu1-32 ura4-D18*	This study
KD2465	*h- 10myc-rad3-nmtT-KanR*	This study
KD2466	*h- 10myc-rad3 (E1369K)-nmtT-KanR*	This study
KD2467	*h+ 10myc-Rad3-nmtT-KanR rad26::ura4*	This study
KD2468	*h- 10myc-rad3 (E1369K)-nmtT-KanR rad26::ura4*	This study
KD2470	*h-10myc-rad3 (E1369K)-nmtT-KanR rad26::Ura4 [pIRT-2L]*	This study
KD2471	*h-10myc-rad3 (E1369K)-nmtT-KanR rad26::ura4 3HA-rad26 [pIRT-2L]*	This study
KD2472	*h- 10myc-rad3 (E1369K)-nmtT-KanR rad26::ura4 3HA-rad26(Δ1-30aa)[pIRT-2L]*	This study
KD2473	*h-10myc-rad3 (E1369K)-nmtT-KanR rad26::ura4 3HA-rad26(KKRK)[pIRT-2L]*	This study
KD2474	*h-10myc-rad3 (E1369K)-nmtT-KanR rad26::ura4 3HA-rad26(F18A)[pIRT-2L]*	This study
KD2728	*h-10myc-rad3 (E1369K)-nmtT-KanR rad26::ura4 3HA-rad26(Δ1-30+KKRK)[pIRT-2L]*	This study
KD2480	*h+ 10myc-Rad3-nmtT:KanR rad26::ura4[pIRT-2L]*	This study
KD2481	*h+ 10myc-Rad3-nmtT:KanR rad26::ura4 3HA-rad26 [pIRT-2L]*	This study

KD2482	*h+ 10myc-Rad3-nmtT:KanR rad26::ura4 3HA-rad26(Δ1-30aa)[pIRT-2L]*	This study
KD2483	*h+ 10myc-Rad3-nmtT:KanR rad26::ura4 3HA-rad26(KKRK)[pIRT-2L]*	This study
KD2484	*h+ 10myc-Rad3-nmtT:KanR rad26::ura4 3HA-rad26(F18A)[pIRT-2L]*	This study
KD2727	*h+ 10myc-Rad3-nmtT:KanR rad26::ura4 3HA-rad26(Δ1-30+KKRK)[pIRT-2L]*	This study
KD2527	*h-10myc-rad3 (E1369K)-nmtTerm:KanR Δrad9::ura4*	This study
KD2528	*h+ 10myc-rad3 (E1369K)-nmtTerm:KanR Δrad1::ura4*	This study
KD2529	*h-10myc-rad3 (E1369K)-nmtTerm:KanR Δrad17::ura4*	This study
KD2515	*h+ 10myc-rad3(E1369K)-nmtT-KanR rad9 (Δ411-426) cds1-6his2HA*	This study
KD2516	*h+ 10myc-Rad3-nmtT-KanR rad9 (Δ411-426) cds1-6his2HA*	This study
KD2587	*h+ 10myc-rad3(E1369K)-nmtT-KanR cds1-6his2HA*	This study
KD2589	*h- 10myc-Rad3-nmtT-KanR cds1-6his2HA*	This study
KD2602	*h+ 10myc-Rad3-nmtTerm:KanR chk1-9myc-2HA6his:ura4 rad9 ((Δ411-426) cds1-6hisHA*	This study
KD2604	*h+ 10myc-rad3 (E1369K)-nmtTerm:KanR chk1-9myc-2HA6his:ura4 rad9 ((Δ411-426) cds1-6hisHA*	This study
KD2517	*h?rad3::ura4 cds1-6hisHA leu1-32 ura4-D18*	This study
KD2518	*h?rad3::ura4 cds1-6hisHA leu1-32 ura4-D18[pIRT-2L]*	This study
KD2819	*h+ 10myc-rad3(F2250L)-nmtT:kanR rad9 (Δ411-426) cds1-6his2HA*	This study
KD2821	*h- 10myc-rad3(F2250L)-nmtT:kanR cds1-6his2HA*	This study
KD2849	*h-10myc-rad3 (E1369K)-nmtTerm:KanR rad9 (Δ411-426) Δmrc1::ura4 leu1:mrc1(T645A+T653A) cds1-6his2HA*	This study
KD2850	*h- 10myc-rad3 (E1369K)-nmtTerm:KanR Δmrc1::ura4 leu1:mrc1(T645A+T653A) cds1-6his2HA*	This study
KD2851	*h+ 10myc-rad3 (E1369K)-nmtTerm:KanR rad9 (Δ411-426) Δmrc1::ura4 cds1-6his2HA*	This study
KD2852	*h+10myc-rad3 (E1369K)-nmtTerm:KanR Δmrc1::ura4 cds1-6his2HA*	This study
KD2859	*h-rad3::ura4 leu1-32 ura4-D18 ade6 [pIRT-2L]*	This study
KD2860	*h-rad3::ura4 leu1-32 ura4-D18 ade6 prom-10myc-rad3(E1369K)[pIRT-2L]*	This study
