# Supplementary material for: A point mutation in the FAT domain constitutively increases the kinase activity of Rad3ATR and bypasses the requirement for 9-1–1 phosphorylation to activate the DNA replication checkpoint
Source: PLoS Genet. 2026 Jun 22;22(6):e1012213. doi: 10.1371/journal.pgen.1012213 (PMC13309046; doi:10.1371/journal.pgen.1012213)
Supplement: S2 Table — (PDF) [file pgen.1012213.s011.pdf]

S2 Table. List of plasmids used in this study

Name	***Description***	Sources
pIRT-2L	*expression vector with LEU2 marker*	Lab stock
pIRT-2U	*expression vector with URA4 marker*	Lab stock
pYJ2012	*Prom-3HA-rad26(SpeI)/LEU2*	Lab stock
pYJ2020	*Prom-3HA-rad26(Δ1-30)/LEU2*	Lab stock
pYJ2043	*Prom-3HA-rad26(K203A-K204A-R205A-K206A)/LEU2*	Lab stock
pYJ2110	*Prom-3HA-rad26(F18A)/LEU2*	Lab stock
pYJ2169	*Prom-3HA-rad26(Δ1-30 K203A-K204A-R205A-K206A)/LEU2*	Lab stock
pYJ1277	*Prom-Suc22-term(BamHI-SalI)/URA4*	Lab stock
pYJ901	*Prom-Rad9(SalI-XmaI/)LEU2*	Lab stock
pYJ1266	*Prom-Cut5(BamHI-XmaI)nmtTERM/URA4*	Lab stock
pYJ1427	*Prom-10myc-rad3/LEU2*	Lab stock
pKD2177	*pGEM7-promo-10myc-rad3-E1369K-nmtTERM-Kan-Rad3C-term*	This study
pKD2176	*pGEM7-promo-10myc-Rad3-nmtTERM-Kan-Rad3C-term*	This study
pKD2206	*Prom-10myc-rad3(E1369K)/LEU2*	This study
