# Supplementary material for: A point mutation in the FAT domain constitutively increases the kinase activity of Rad3ATR and bypasses the requirement for 9-1–1 phosphorylation to activate the DNA replication checkpoint
Source: PLoS Genet. 2026 Jun 22;22(6):e1012213. doi: 10.1371/journal.pgen.1012213 (PMC13309046; doi:10.1371/journal.pgen.1012213)
Supplement: S3 Table — (PDF) [file pgen.1012213.s012.pdf]

S3 Table. List of primers used in this study

Name	Sequence (5´ -> 3´)	Note
KanR-f	GTATGTGAATGCTGGTCGC	Colony PCR
rad3-N-int-primer	GAAAAACGCAGGCCATCCA	
Rad3 term b2	CAAGAAATTGAACAACTCAGC	
Rad9(t)r	TGTCACAAGCACCTGTAAATGTTC	Sequencing Rad9 C-terminus
Rad9(821-838)f	TCCCAGGAAAACCGATAC	
Rad3(253-232)r	GGGCTTCTTTTAGCATCTTAGG	Sequencing Rad3
Rad3(733-750)f	CCTCTCTTTACAGCGTGG	
Rad3(1193-1176)r	ATTTCGCCAAAAGCACGG	
Rad3(1351-1370)f	AACAACTTAGATGAACCGTC	
Rad3(2022-2039)f	TCAAGGCATTGGTCTCAG	
Rad3(2746-2728)r	GTAATCCAAGTTTGACTGC	
Rad3(3227-3210)r	TGGTCCAGAACTTCGTTG	
Rad3(3800-3790)r	ACCTCTTGGTCTTTCACTAC	
Rad3(4409-4392)r	TAACAACTCAAAGCCGAG	
Rad3(4931-4914)r	GTGGACAGGATTTGGTGC	
Rad3(5616-5597)r	CGAAGAGATAATGTGTTCAC	
Rad3(5335-5353)f	TCAGAATGTGAGAATACGC	
Rad3(5785-5806)f	TATCCTGGGGAGACGCTATGGC	
Rad3(6325-6342)f	CAAGAAGCGAACAGAAGG	
P1	Biotin-TGCAACAACTTTTTTCGCTCCCGATAGTGAGTCGTA TTTTTAGCTGAGCCAGGTTTCCCCGATTTCCAGAC	Pull-down
P2	GTCTGGAAATCGGGGAAACCTGGCTCAGCTAAAAAATACG ACTCACTATCGGGAGCGAAAAAAGTTGTTGCA	Pull-down
