# Supplementary material for: A point mutation in the FAT domain constitutively increases the kinase activity of Rad3ATR and bypasses the requirement for 9-1–1 phosphorylation to activate the DNA replication checkpoint
Source: PLoS Genet. 2026 Jun 22;22(6):e1012213. doi: 10.1371/journal.pgen.1012213 (PMC13309046; doi:10.1371/journal.pgen.1012213)
Supplement: Attachment — Submitted filename: Response to Reviewers Comments.docx [file pgen.1012213.s014.docx]

**Response to Reviewers’ Comments**

Note: The reviewers’ comments are in **blue** and the responses in **black**.

Reviewer #1: This manuscript by Dev and colleagues identifies a charge-reversing mutation (E1369K) in the FAT domain of Rad3 in fission yeast that enhances kinase activity and allows bypass of Rad9 phosphorylation–dependent checkpoint activation. The study addresses an important question in ATR/Rad3 signaling, and the genetic and biochemical approaches are generally well executed with multiple complementary readouts. From the perspective of ATR biology, the finding that kinase activity can be elevated with reduced reliance on canonical activators is interesting and potentially relevant beyond yeast systems. However, I have a few concerns regarding interpretation and clarity.

The authors describe Rad3 E1369K as constitutively active; while the data support increased kinase activity and reduced dependence on Rad9 phosphorylation–dependent activation, it may be helpful to clarify what is meant by “constitutive” in this context, as signaling still depends on Rad26-mediated recruitment, the 9 1 1 complex, and Mrc1. More precise wording (e.g., hyperactive or partially bypassing canonical activation) may help avoid overinterpretation.

**Response:** We greatly appreciate the positive and constructive suggestions and comments from both reviewers. Regarding the Rad3-E1369K mutant, both “constitutively active” and “hyperactive” describe its characteristics. After careful consideration and discussion, we have decided to use the term “constitutively active” for the following reasons: We know Rad3 is active in HU-treated cells, ie, in an active state, and the activated Rad3, when IPed from the HU-treated cells, becomes inactive in the in vitro kinase assay, suggesting that active Rad3 returns to the inactive state when purified from the cells. On the contrary, the IPed Rad3-E1369K always shows higher kinase activity, whether IPed from HU-treated or untreated cells, suggesting that mutant Rad3 cannot return to the inactive state like wt Rad3; i.e., the mutation locks Rad3 in a constitutively active state, whether partially or fully active. On the other hand, we do not know whether Rad3-E1369K is “hyperactive”, because so far, we do not know the level of a fully active wt Rad3. Whenever wt Rad is IPed out, it returns to the inactive state in the in vitro kinase assay. We have previously shown that addition of purified Rad4 or Rad4 C-terminus did not activate Rad3 or budding yeast Mec1 like Dpb11 or TopBP1 (PMID: 24663817).

In addition, the distinction between activation and recruitment could be more clearly articulated, as Rad26 functions primarily in recruitment rather than direct activation.

**Response:** Thanks for the suggestion. This issue has been fixed (see lines 313-314).

The proposed FAT-domain allosteric activation model is intriguing but remains somewhat speculative and could be framed more cautiously.

**Response:** We appreciate the suggestion and have reworded the allosteric mechanism more cautiously (see lines 30-33 and lines 483-490).

Minor issues include occasional unclear phrasing (e.g., ambiguous “it”) and a few figure reference errors (e.g., Fig. 2D vs S2D and Fig. 4A vs 5A). Overall, this is a solid and interesting study, but clarifying these points would improve conceptual precision and presentation.

**Response:** We appreciate the positive comment. These minor issues have all been fixed.

Reviewer #2: The manuscript addresses an important unresolved question in checkpoint signaling: the mechanistic basis of Rad3/ATR activation during the replication checkpoint response. Using an unbiased suppressor screen in rad9 phospho-mutants, the authors identify a gain-of-function mutation in the FAT domain of Rad3 (E1369K) that restores Cds1 phosphorylation and improves resistance to replication stress. The study combines suppressor genetics, checkpoint signaling assays, and in vitro kinase assays to support a model in which the FAT domain can allosterically regulate Rad3 kinase activity independently of the canonical PRD-based activation mechanism. Overall, I find the study mechanistically interesting, logically developed, and suitable for publication after a revision.

The work has several notable strengths. First, the suppressor screen is elegant and unbiased, making the identification of a Rad3 FAT-domain mutation particularly compelling. Second, the authors convincingly demonstrate that the E1369K mutation restores checkpoint signaling rather than merely conferring nonspecific stress resistance, as evidenced by restoration of Cds1 phosphorylation. Third, the authors directly address an important alternative explanation—enhanced Rad3 recruitment—using co-IP and DNA pull-down assays, which argue against increased Rad3–Rad26 association or localization as the primary cause of the phenotype. The biochemical kinase assays further strengthen the study by demonstrating elevated intrinsic kinase activity of Rad3-E1369K. Finally, comparison with the Rad3-F2250L mutation (equivalent to Mec1-F2244L) strengthens the mechanistic interpretation by showing that not all activating mutations behave equivalently.

My main concern relates to interpretation rather than the core experimental findings. The data convincingly demonstrate that the FAT domain can regulate Rad3 kinase activity allosterically and that the E1369K mutation can bypass canonical checkpoint activation requirements. However, the broader conclusion that physiological Rad3 activation involves FAT-domain allostery appears somewhat stronger than directly supported by the current evidence. Specifically, while the E1369K mutation generates a constitutively hyperactive form of Rad3, the study does not directly demonstrate that endogenous checkpoint activation engages the same FAT-domain–mediated conformational mechanism. In particular, the work does not establish a direct link between Rad9/Rad4-mediated signaling and FAT-domain conformational changes in the native pathway. Given the technical challenges of probing such mechanisms in large PIKK-family kinases, I do not consider additional major experiments essential for publication. However, the manuscript would benefit from moderating statements in the Discussion and framing FAT-domain allostery as a plausible contributor to physiological Rad3 activation, rather than a definitively established mechanism.

**Response:** We greatly appreciate the reviewer’s careful reading of the manuscript and the thoughtful insights. We agree with the review that the activation mechanism of Rad3 in the drug-treated cells may not follow the same “activation mechanism” caused by the E1369K mutation, as the structural evidence of the activation mechanism of Rad3 (or ATR/Mec1) is still lacking. According to the current model, Rad3 activation likely involves lifting the autoinhibitory PRD in the kinase domain, whether it is through the conformational change in the FAT domain or other mechanisms, such as binding to a yet to be identified Rad3 activator. We appreciate this suggestion from the reviewer and the editors and add more discussion to clarify this issue (see lines 483 - 490).

Related to this point, it would be helpful to more clearly distinguish between the constitutive activation produced by the E1369K mutation and the conformational state induced during physiological checkpoint activation. These states may share features but are not necessarily identical.

**Response:** We agree with the reviewer’s great insightful comment. Since the structural evidence is still lacking, it is unclear whether the mutation-induced active Rad3 shares the same active state induced endogenously (see lines 486-488).

Minor comments:
1. The comparison between Rad3-E1369K and Rad3-F2250L is informative and strengthens the manuscript; a brief discussion of why equivalent Mec1 and Rad3 mutations may behave differently across species would add useful context.

**Response:** Following the suggestion, we briefly explain the potential reason for the difference (see lines 460-466).

2. The suggestion that the Rad9 C-terminus may exert both positive and negative regulatory effects on Rad3 activity is intriguing; this could be more explicitly framed as a working model.

**Response:** We agree with the reviewer that it is a surprise to see the Rad9 C-terminus can also negatively regulate Rad3 kinase activity. Following the suggestion, we provide a few potential insights in the discussion (515-517).

3. The manuscript is generally well written, and the experimental logic is easy to follow. The schematic overview figures are particularly helpful given the complexity of checkpoint nomenclature across species. Overall, this is a strong mechanistic genetics study that makes a meaningful contribution to understanding ATR/Rad3 regulation. With moderation of claims regarding physiological FAT-domain allostery, I believe the manuscript will be suitable for publication.

**Response:** We greatly appreciate the positive comments from this reviewer.
