# Supplementary material for: A point mutation in the FAT domain constitutively increases the kinase activity of Rad3ATR and bypasses the requirement for 9-1–1 phosphorylation to activate the DNA replication checkpoint
Source: PLoS Genet. 2026 Jun 22;22(6):e1012213. doi: 10.1371/journal.pgen.1012213 (PMC13309046; doi:10.1371/journal.pgen.1012213)
Supplement: Peer review history

# Peer review: Decision Letter 0

**DOI:** 10.1371/journal.pgen.1012213.r001

**Authors:** Bruce Clurman, Pablo Wappner

27 May 2026

PGENETICS-D-26-00341

A point mutation in the FAT domain constitutively increases the kinase activity of Rad3ATR and bypasses the requirement for 9-1-1 phosphorylation to activate the DNA replication checkpoint

PLOS Genetics

Dear Dr. Xu,

Thank you for submitting your manuscript to PLOS Genetics. After careful consideration, we feel that it has merit but does not fully meet PLOS Genetics's publication criteria as it currently stands. Therefore, we invite you to submit a revised version of the manuscript that addresses the points raised during the review process.

Please submit your revised manuscript within by Jun 26 2026 11:59PM. If you will need more time than this to complete your revisions, please reply to this message or contact the journal office at plosgenetics@plos.org. When you're ready to submit your revision, log on to https://www.editorialmanager.com/pgenetics/ and select the 'Submissions Needing Revision' folder to locate your manuscript file.

Please include the following items when submitting your revised manuscript:

* A letter that responds to each point raised by the editor and reviewer(s). You should upload this letter as a separate file labeled 'Response to Reviewers'. This file does not need to include responses to formatting updates and technical items listed in the 'Journal Requirements' section below.

* A marked-up copy of your manuscript that highlights changes made to the original version. You should upload this as a separate file labeled 'Revised Manuscript with Track Changes'.

* An unmarked version of your revised paper without tracked changes. You should upload this as a separate file labeled 'Manuscript'.

If you would like to make changes to your financial disclosure, competing interests statement, or data availability statement, please make these updates within the submission form at the time of resubmission. Guidelines for resubmitting your figure files are available below the reviewer comments at the end of this letter.

As the corresponding author, your ORCID iD is verified in the submission system and will appear in the published article. PLOS supports the use of ORCID, and we encourage all coauthors to register for an ORCID iD and use it as well. Please encourage your coauthors to verify their ORCID iD within the submission system before final acceptance, as unverified ORCID iDs will not appear in the published article. Only the individual author can complete the verification step; PLOS staff cannot verify ORCID iDs on behalf of authors.

We look forward to receiving your revised manuscript.

Kind regards,

Bruce E. Clurman

Academic Editor

PLOS Genetics

Pablo Wappner

Section Editor

PLOS Genetics

Aimée Dudley

Editor-in-Chief

PLOS Genetics

Anne Goriely

Editor-in-Chief

PLOS Genetics

**Additional Editor Comments:**

Both Reviewers found your paper to be suitable for publication but also had some comments that require responses. Reviewer 2 in particular raised a number of points that should be addressed. The most significant is the issue of whether endogenous checkpoint activation engages the same FAT-domain–mediated conformational mechanism. This is an important point: the Reviewer acknowledged the technical challenges in addressing this question and instead asked for this conclusion be clearly qualified, and for the uncertainty around the endogenous pathways to be clearly stated.

**Journal Requirements:**

If the reviewer comments include a recommendation to cite specific previously published works, please review and evaluate these publications to determine whether they are relevant and should be cited. There is no requirement to cite these works unless the editor has indicated otherwise.

1) Some material included in your submission may be copyrighted. According to PLOS’s copyright policy, authors who use figures or other material (e.g., graphics, clipart, maps) from another author or copyright holder must demonstrate or obtain permission to publish this material under the Creative Commons Attribution 4.0 International (CC BY 4.0) License used by PLOS journals. Please closely review the details of PLOS’s copyright requirements here: PLOS Licenses and Copyright. If you need to request permissions from a copyright holder, you may use PLOS's Copyright Content Permission form.

Please respond directly to this email and provide any known details concerning your material's license terms and permissions required for reuse, even if you have not yet obtained copyright permissions or are unsure of your material's copyright compatibility. Once you have responded and addressed all other outstanding technical requirements, you may resubmit your manuscript within Editorial Manager.

Potential Copyright Issues:

i) Please confirm (a) that you are the photographer of S1, or (b) provide written permission from the photographer to publish the photo(s) under our CC BY 4.0 license.

**Reviewers' comments:**

Reviewer's Responses to Questions

**Comments to the Authors:**

**Please note here if the review is uploaded as an attachment.**

Reviewer #1: This manuscript by Dev and colleagues identifies a charge-reversing mutation (E1369K) in the FAT domain of Rad3 in fission yeast that enhances kinase activity and allows bypass of Rad9 phosphorylation–dependent checkpoint activation. The study addresses an important question in ATR/Rad3 signaling, and the genetic and biochemical approaches are generally well executed with multiple complementary readouts. From the perspective of ATR biology, the finding that kinase activity can be elevated with reduced reliance on canonical activators is interesting and potentially relevant beyond yeast systems. However, I have a few concerns regarding interpretation and clarity. The authors describe Rad3 E1369K as constitutively active; while the data support increased kinase activity and reduced dependence on Rad9 phosphorylation–dependent activation, it may be helpful to clarify what is meant by “constitutive” in this context, as signaling still depends on Rad26-mediated recruitment, the 9 1 1 complex, and Mrc1. More precise wording (e.g., hyperactive or partially bypassing canonical activation) may help avoid overinterpretation. In addition, the distinction between activation and recruitment could be more clearly articulated, as Rad26 functions primarily in recruitment rather than direct activation. The proposed FAT-domain allosteric activation model is intriguing but remains somewhat speculative and could be framed more cautiously. Minor issues include occasional unclear phrasing (e.g., ambiguous “it”) and a few figure reference errors (e.g., Fig. 2D vs S2D and Fig. 4A vs 5A). Overall, this is a solid and interesting study, but clarifying these points would improve conceptual precision and presentation.

Reviewer #2: The manuscript addresses an important unresolved question in checkpoint signaling: the mechanistic basis of Rad3/ATR activation during the replication checkpoint response. Using an unbiased suppressor screen in rad9 phospho-mutants, the authors identify a gain-of-function mutation in the FAT domain of Rad3 (E1369K) that restores Cds1 phosphorylation and improves resistance to replication stress. The study combines suppressor genetics, checkpoint signaling assays, and in vitro kinase assays to support a model in which the FAT domain can allosterically regulate Rad3 kinase activity independently of the canonical PRD-based activation mechanism. Overall, I find the study mechanistically interesting, logically developed, and suitable for publication after a revision.

The work has several notable strengths. First, the suppressor screen is elegant and unbiased, making the identification of a Rad3 FAT-domain mutation particularly compelling. Second, the authors convincingly demonstrate that the E1369K mutation restores checkpoint signaling rather than merely conferring nonspecific stress resistance, as evidenced by restoration of Cds1 phosphorylation. Third, the authors directly address an important alternative explanation—enhanced Rad3 recruitment—using co-IP and DNA pull-down assays, which argue against increased Rad3–Rad26 association or localization as the primary cause of the phenotype. The biochemical kinase assays further strengthen the study by demonstrating elevated intrinsic kinase activity of Rad3-E1369K. Finally, comparison with the Rad3-F2250L mutation (equivalent to Mec1-F2244L) strengthens the mechanistic interpretation by showing that not all activating mutations behave equivalently.

My main concern relates to interpretation rather than the core experimental findings. The data convincingly demonstrate that the FAT domain can regulate Rad3 kinase activity allosterically and that the E1369K mutation can bypass canonical checkpoint activation requirements. However, the broader conclusion that physiological Rad3 activation involves FAT-domain allostery appears somewhat stronger than directly supported by the current evidence.

Specifically, while the E1369K mutation generates a constitutively hyperactive form of Rad3, the study does not directly demonstrate that endogenous checkpoint activation engages the same FAT-domain–mediated conformational mechanism. In particular, the work does not establish a direct link between Rad9/Rad4-mediated signaling and FAT-domain conformational changes in the native pathway. Given the technical challenges of probing such mechanisms in large PIKK-family kinases, I do not consider additional major experiments essential for publication. However, the manuscript would benefit from moderating statements in the Discussion and framing FAT-domain allostery as a plausible contributor to physiological Rad3 activation, rather than a definitively established mechanism.

Related to this point, it would be helpful to more clearly distinguish between the constitutive activation produced by the E1369K mutation and the conformational state induced during physiological checkpoint activation. These states may share features but are not necessarily identical.

Minor comments:

1. The comparison between Rad3-E1369K and Rad3-F2250L is informative and strengthens the manuscript; a brief discussion of why equivalent Mec1 and Rad3 mutations may behave differently across species would add useful context.

2. The suggestion that the Rad9 C-terminus may exert both positive and negative regulatory effects on Rad3 activity is intriguing; this could be more explicitly framed as a working model.

3. The manuscript is generally well written, and the experimental logic is easy to follow. The schematic overview figures are particularly helpful given the complexity of checkpoint nomenclature across species.

Overall, this is a strong mechanistic genetics study that makes a meaningful contribution to understanding ATR/Rad3 regulation. With moderation of claims regarding physiological FAT-domain allostery, I believe the manuscript will be suitable for publication.

**********

**Have all data underlying the figures and results presented in the manuscript been provided?**

Large-scale datasets should be made available via a public repository as described in the *PLOS Genetics*  data availability policy, and numerical data that underlies graphs or summary statistics should be provided in spreadsheet form as supporting information.

Reviewer #1: Yes

Reviewer #2: Yes

**********

PLOS authors have the option to publish the peer review history of their article (what does this mean?). If published, this will include your full peer review and any attached files.

If you choose “no”, your identity will remain anonymous but your review may still be made public.

**Do you want your identity to be public for this peer review?**  For information about this choice, including consent withdrawal, please see our Privacy Policy.

Reviewer #1: No

Reviewer #2: No

**Figure resubmission:**

While revising your submission, we strongly recommend that you use PLOS’s NAAS tool (https://ngplosjournals.pagemajik.ai/artanalysis) to test your figure files. NAAS can convert your figure files to the TIFF file type and meet basic requirements (such as print size, resolution), or provide you with a report on issues that do not meet our requirements and that NAAS cannot fix.

After uploading your figures to PLOS’s NAAS tool - https://ngplosjournals.pagemajik.ai/artanalysis, NAAS will process the files provided and display the results in the "Uploaded Files" section of the page as the processing is complete. If the uploaded figures meet our requirements (or NAAS is able to fix the files to meet our requirements), the figure will be marked as "fixed" above. If NAAS is unable to fix the files, a red "failed" label will appear above. When NAAS has confirmed that the figure files meet our requirements, please download the file via the download option, and include these NAAS processed figure files when submitting your revised manuscript.

**Reproducibility:**

To enhance the reproducibility of your results, we recommend that authors deposit laboratory protocols in protocols.io, where a protocol can be assigned its own identifier (DOI) such that it can be cited independently in the future. Additionally, PLOS ONE offers an option to publish peer-reviewed clinical study protocols. Read more information on sharing protocols at https://plos.org/protocols?utm_medium=editorial-email&utm_source=authorletters&utm_campaign=protocols

# Author comment: Author response to Decision Letter 1

**DOI:** 10.1371/journal.pgen.1012213.r002

9 Jun 2026

Attachment Submitted filename: Response to Reviewers Comments.docx

# Editor report: Decision Letter 1

**DOI:** 10.1371/journal.pgen.1012213.r003

**Authors:** Bruce Clurman, Pablo Wappner, Bruce Clurman, Pablo Wappner

15 Jun 2026

Dear Dr Xu,

We are pleased to inform you that your manuscript entitled "A point mutation in the FAT domain constitutively increases the kinase activity of Rad3ATR and bypasses the requirement for 9-1-1 phosphorylation to activate the DNA replication checkpoint" has been editorially accepted for publication in PLOS Genetics. Congratulations!

Before your submission can be formally accepted and sent to production you will need to complete our formatting changes, which you will receive in a follow up email. Please be aware that it may take several days for you to receive this email; during this time no action is required by you. Please note: the accept date on your published article will reflect the date of this provisional acceptance, but your manuscript will not be scheduled for publication until the required changes have been made.

Once your paper is formally accepted, an uncorrected proof of your manuscript will be published online ahead of the final version, unless you’ve already opted out via the online submission form. If, for any reason, you do not want an earlier version of your manuscript published online or are unsure if you have already indicated as such, please let the journal staff know immediately at plosgenetics@plos.org.

In the meantime, please log into Editorial Manager at https://www.editorialmanager.com/pgenetics/, click the "Update My Information" link at the top of the page, and update your user information to ensure an efficient production and billing process. Note that PLOS requires an ORCID iD for all corresponding authors. Therefore, please ensure that you have an ORCID iD and that it is validated in Editorial Manager. To do this, go to ‘Update my Information’ (in the upper left-hand corner of the main menu), and click on the Fetch/Validate link next to the ORCID field.  This will take you to the ORCID site and allow you to create a new iD or authenticate a pre-existing iD in Editorial Manager.

If you have a press-related query, or would like to know about making your underlying data available (as you will be aware, this is required for publication), please see the end of this email. If your institution or institutions have a press office, please notify them about your upcoming article at this point, to enable them to help maximise its impact. Inform journal staff as soon as possible if you are preparing a press release for your article and need a publication date.

Thank you again for supporting open-access publishing; we are looking forward to publishing your work in PLOS Genetics!

Yours sincerely,

Bruce E. Clurman

Academic Editor

PLOS Genetics

Pablo Wappner

Section Editor

PLOS Genetics

Aimée Dudley

Editor-in-Chief

PLOS Genetics

Anne Goriely

Editor-in-Chief

PLOS Genetics

www.plosgenetics.org

BlueSky: @plos.bsky.social

----------------------------------------------------

Comments from the reviewers (if applicable):

These revisions appropriately address the reviews.

----------------------------------------------------

**Data Deposition**

If you have submitted a Research Article or Front Matter that has associated data that are not suitable for deposition in a subject-specific public repository (such as GenBank or ArrayExpress), one way to make that data available is to deposit it in the Dryad Digital Repository. As you may recall, we ask all authors to agree to make data available; this is one way to achieve that. A full list of recommended repositories can be found on our website.

The following link will take you to the Dryad record for your article, so you won't have to re‐enter its bibliographic information, and can upload your files directly:

http://datadryad.org/submit?journalID=pgenetics&manu=PGENETICS-D-26-00341R1

More information about depositing data in Dryad is available at http://www.datadryad.org/depositing. If you experience any difficulties in submitting your data, please contact help@datadryad.org for support.

Additionally, please be aware that our data availability policy requires that all numerical data underlying display items are included with the submission, and you will need to provide this before we can formally accept your manuscript, if not already present.

----------------------------------------------------

**Press Queries**

If you or your institution will be preparing press materials for this manuscript, or if you need to know your paper's publication date for media purposes, please inform the journal staff as soon as possible so that your submission can be scheduled accordingly. Your manuscript will remain under a strict press embargo until the publication date and time. This means an early version of your manuscript will not be published ahead of your final version. PLOS Genetics may also choose to issue a press release for your article. If there's anything the journal should know or you'd like more information, please get in touch via plosgenetics@plos.org.

# Editor report: Acceptance letter

**DOI:** 10.1371/journal.pgen.1012213.r004

**Authors:** Bruce Clurman, Pablo Wappner, Bruce Clurman, Pablo Wappner

PGENETICS-D-26-00341R1

A point mutation in the FAT domain constitutively increases the kinase activity of Rad3ATR and bypasses the requirement for 9-1-1 phosphorylation to activate the DNA replication checkpoint

Dear Dr Xu,

We are pleased to inform you that your manuscript entitled "A point mutation in the FAT domain constitutively increases the kinase activity of Rad3ATR and bypasses the requirement for 9-1-1 phosphorylation to activate the DNA replication checkpoint" has been formally accepted for publication in PLOS Genetics! Your manuscript is now with our production department and you will be notified of the publication date in due course.

The corresponding author will soon be receiving a typeset proof for review, to ensure errors have not been introduced during production. Please review the PDF proof of your manuscript carefully, as this is the last chance to correct any errors. Please note that major changes, or those which affect the scientific understanding of the work, will likely cause delays to the publication date of your manuscript.

Soon after your final files are uploaded, unless you have opted out or your manuscript is a front-matter piece, the early version of your manuscript will be published online. The date of the early version will be your article's publication date. The final article will be published to the same URL, and all versions of the paper will be accessible to readers.

For Research Articles, you will receive an invoice from PLOS for your publication fee after your manuscript has reached the completed accept phase. If you receive an email requesting payment before acceptance or for any other service, this may be a phishing scheme. Learn how to identify phishing emails and protect your accounts at https://explore.plos.org/phishing.

Thank you again for supporting PLOS Genetics and open-access publishing. We are looking forward to publishing your work!

With kind regards,

Janani Seenivasan

PLOS Genetics

On behalf of:

The PLOS Genetics Team

Carlyle House, Carlyle Road, Cambridge CB4 3DN | United Kingdom

plosgenetics@plos.org | +44 (0) 1223-442823

plosgenetics.org | Twitter: @PLOSGenetics
